# MEK1/2 inhibition rescues neurodegeneration by TFEB-mediated activation of autophagic lysosomal function in a model of Alzheimer’s Disease

**DOI:** 10.1038/s41380-022-01713-5

**Published:** 2022-08-10

**Authors:** Yoon Sun Chun, Mi-Yeon Kim, Sun-Young Lee, Mi Jeong Kim, Tae-Joon Hong, Jae Kyong Jeon, Dulguun Ganbat, Hyoung Tae Kim, Sang Seong Kim, Tae-In Kam, Sungho Han

**Affiliations:** 1 Genuv Inc., Seoul, 04520 Republic of Korea; 2 College of Pharmacy, Hanyang University ERICA, Gyeonggi-do, 15588 Republic of Korea; 3 Neuroregeneration and Stem Cell Programs, Institute for Cell Engineering, Johns Hopkins University School of Medicine, Baltimore, MD 21205 USA; 4 Department of Neurology, Johns Hopkins University School of Medicine, Baltimore, MD 21205 USA; 5 Present Address: Department of photonics and nanoelectronics, Hanyang University ERICA, Gyeonggi-do, 15588 Republic of Korea

**Keywords:** Molecular biology, Neuroscience, Cell biology

## Abstract

Alzheimer’s Disease (AD) is a progressive neurodegenerative disorder, which is characterized by cognitive deficit due to synaptic loss and neuronal death. Extracellular amyloid β plaques are one of the pathological hallmarks of AD. The autophagic lysosomal pathway is the essential mechanism to maintain cellular homeostasis by driving clearance of protein aggregates and is dysfunctional in AD. Here, we showed that inhibiting MEK/ERK signaling using a clinically available MEK1/2 inhibitor, trametinib (GSK1120212, SNR1611), induces the protection of neurons through autophagic lysosomal activation mediated by transcription factor EB (TFEB) in a model of AD. Orally administered trametinib recovered impaired neural structures, cognitive functions, and hippocampal long-term potentiation (LTP) in 5XFAD mice. Trametinib also reduced Aβ deposition via induction of autophagic lysosomal activation. RNA-sequencing analysis revealed upregulation of autophagic lysosomal genes by trametinib administration. In addition, trametinib inhibited TFEB phosphorylation at Ser142 and promoted its nuclear translocation, which in turn induced autophagic lysosomal related genes, indicating that trametinib activates the autophagic lysosomal process through TFEB activation. From these observations, we concluded that MEK inhibition provides neuronal protection from the Aβ burden by increasing autophagic lysosomal activity. Thus, MEK inhibition may be an effective therapeutic strategy for AD.

## Introduction

Alzheimer’s disease (AD) is a neurodegenerative disorder that typically begins with memory loss and cognitive impairment, accompanied by neuronal and synaptic loss in the cerebral cortex and the hippocampus. Its main features are the accumulation of extracellular β-amyloid (Aβ) plaques, intracellular neurofibrillary tangles composed of hyperphosphorylated tau [1, 2]. These aberrant accumulations are implicated in pathophysiological processes, including mitochondrial and lysosomal dysfunction, oxidative stress, neuroinflammation, and more [3–5].

The autophagic lysosomal pathway is one of the key mechanisms to maintain cellular homeostasis by removing cytotoxic protein aggregates, long-lived proteins, dysfunctional cellular organelles, and invaded pathogens [6, 7]. Autophagy is involved in neuronal development as well as functions and survival of neurons, and defects in autophagy have been associated with neurodegenerative diseases [8]. Mice lacking essential autophagy-related genes, specifically *Atg5* or *Atg7*, in the central nervous system showed progressive neuronal loss and accumulation of abnormal proteins, which eventually lead to neurodegeneration [9, 10]. Autophagic vacuoles accumulate within neurites and synapses in AD brains, suggesting defects in autophagosome maturation and fusion with a lysosome [11, 12]. In addition, autophagy induced by mTOR inhibition lowers levels of amyloid β and phosphorylated tau and thus ameliorates synaptic and cognitive deficits in the AD animal model [13–15]. Therefore, neuronal autophagy seems to play a critical role in synapse plasticity required for learning and memory [16], and the upregulation of autophagy is one promising strategy to eliminate AD-causative proteins and recover neuronal function.

The mitogen-activated kinase pathway (Ras/Raf/MEK/ERK pathway) mediates intracellular signaling involved in cell proliferation, survival, and death [17, 18]. Emerging evidence has shown the implication of MEK/ERK pathway in the pathogenesis of AD [19–21], while molecular mechanisms by which it contributes to AD pathogenesis are not fully understood. Phospho-MEK1 is accumulated in the gray matter of the temporal cortex from AD patients [22]. ERK is activated in the brain of AD mouse models [23, 24] and cerebrospinal fluid (CSF) from AD patients [25]. Furthermore, Aβ42 fibrils cause abnormal tau phosphorylation and dystrophic neurite production through ERK activation [20, 26]. Previous reports have shown that ERK signaling modulates the expression of autophagy and lysosomal genes by transcription factor EB (TFEB) activity [27], and TFEB accelerates to reduce Aβ generation through lysosomal degradation [28]. However, a therapeutic agent that could selectively inhibit the MEK/ERK pathway has not been evaluated in AD treatment. Trametinib (GSK1120212, SNR1611) is a US Food and Drug Administration-approved anti-cancer drug for melanoma patients to inhibit MEK1 and MEK2 simultaneously (MEK1/2 inhibitor) [29]. Here, we tested the therapeutic efficacy of trametinib in 5XFAD mice that co-overexpress human amyloid precursor protein (APP) and presenilin 1 harboring five familial AD mutations [30] and explored the mechanisms of MEK inhibition in the Aβ-associated autophagic lysosomal activity. Upon oral administration, trametinib rescued cognitive functions and hippocampal long-term potentiation (LTP) and reduced Aβ deposition via inducing autophagic lysosomal degradation. We showed that trametinib elevated autophagic activity by regulating the expression of autophagy and lysosome-related genes through TFEB activation in vivo and in vitro. Therefore, we conclude that MEK inhibition by activating autophagic and lysosomal clearance of Aβ is an effective therapeutic approach for AD.

## Materials and methods

### Animals

B6SJL-Tg (APPSwFlLon, PSEN1*M146L*L286V) (5XFAD) and age-matched WT (B6SJLF1/J) mice were purchased from the Jackson Laboratory and ICR mice were obtained from OrientBio Inc. Experimental procedures were performed according to protocols approved by the Institutional Animal Care and Use Committee (IACUC) of KPCLab (approved number: P171011). C57BL/6 mice were obtained from OrientBio Inc., and compliance with relevant ethical regulations and animal procedures were reviewed and approved by Seoul National University Hospital IACUC (approved number: 16-0043-c1a0).

### Trametinib treatment

Trametinib (MedChemExpress) was micronized and suspended in the vehicle containing 5% mannitol, 1.5% hydroxypropyl methylcellulose, and 0.2% sodium lauryl sulfate. For pharmacokinetic analysis, 0.05, 0.2, and 0.8 mg/kg of trametinib were orally administered to 7-week-old ICR mice (*n* = 5 per group) as a single administration. For pharmacodynamic analysis and RNA sequencing, 0.1 mg/kg/day of trametinib was orally administered to 6-week-old C57BL/6 mice for 1–4 weeks (*n* = 3 per group). Mice were sacrificed at each identical time point. 5XFAD mice (male, *n* = 7–10 per group) received vehicle or 0.1 mg/kg of trametinib for 10 weeks by oral gavage once a day. All the mice were sacrificed by the perfusion method.

### Whole cell RNA sequencing

RNA was isolated from mouse whole brains, and cDNA libraries were prepared using the TruSeq Stranded mRNA Prep Kit (Illumina) [31]. The libraries were sequenced on the Illumina NextSeq500 platform, and the reads were mapped to the reference Mouse mm10 genome using Tophat v2.0.13. Total 24,532 genes were mapped to the mouse transcriptome, and the genes which were not read in at least one sample were removed. There was a total of 15,727 genes after removing genes with 0 counts. To define differentially expressed genes (DEG) using DESeq2 R library, we set up a stringent statistic cutoff of log2 fold change (FC) of ≥1.1 or ≤ −1.1 and a false discovery rate (FDR)<0.05. Gene ontology was performed with the biological process using the Panther database. The significance threshold for analyses was set to 0.05 using Fisher’s exact test-adjusted *p*-values. The per-gene z-score of calculated from log10 FPKM was represented by Morpheus heatmap from autophagic lysosomal-related genes.

### Behavioral test

#### Y-maze test

Mice were placed in the center of the Y-maze, and their activity was recorded for 3 min. The Y-maze is a three-arm maze with 120^◦^ angles between each arm (40 cm long × 15 cm high). Video tracking was performed using Smart video tracking software (Panlab), and the order and number of entries into each arm were recorded. Spontaneous alternation was counted when a mouse made successive entries into the three arms without visiting a previous arm. The experimenters were blinded to treatment condition and randomized.

#### Novel object recognition test

Mice were habituated in an empty open field arena (40 cm × 40 cm). For the training trial, mice were placed in an open field arena with two identical objects for 10 min each. The next day, the test trial was performed for 10 min with one of the two familiar objects replaced with a new one. Video tracking was performed using Smart video tracking software (Panlab), and recognition of familiar and novel objects was calculated as the percentage of time spent on new objects out of the time spent on exploring all objects.

### Electrophysiology

#### Brain slice preparation

High sucrose artificial cerebrospinal fluids (ACSF) were prepared as 0.5 mM CaCl_2_, 2.5 mM KCl, 1.25 mM NaH_2_PO_4_, 5 mM MgSO_4_, 205 mM Sucrose, 5 mM HEPES, 10 mM Glucose, 26 mM NaHCO_3_ (pH = 7.3-7.4, mOsm = 300–310). Recording ACSF was prepared as 126 mM NaCl, 3.5 mM KCl, 1.25 mM NaH_2_PO_4_, 1.6 mM CaCl_2_, 1.2 mM MgSO_4_, 10 mM Glucose, 26 mM NaHCO_3_, 5 mM HEPES (pH = 7.3–7.4, mOsm = 300–310). ACSFs were freshly prepared daily as required. High sucrose ACSF was maintained over ice and saturated by gas infusion of 95% O_2_/5% CO_2_ for at least 20 min. The brain was harvested quickly, in less than 4 min, and chilled for 2 min in pre-oxygenated high sucrose ASCF. Hippocampal sections were coronally sectioned to 300 μm by VF-200 vibratome (Precisionary instrument). For incubation of the slices, they were submerged over nylon mesh in 95% O_2_/5% CO_2_ oxygenated ASCF for 30 min at 32–34 °C and incubated for an additional 30 min at room temperature before first recording.

#### LTP recording with whole-cell Patch clamp

The recording slice was perfused for 30 min in the oxygenated ACSF at 2 ml/min at 28–30 °C in the patch clamp chamber before starting the experiment. We used 4–8 MΩ borosilicate capillary glass electrodes (A-M Systems) pulled from Micropipette puller P-1000 (Sutter Instrument). The intracellular solution consisted of 140 mM K-gluconate, 10 mM KCl, 1 mM EGTA, 10 mM HEPES, 4 mM Na_2_ATP, 0.3 mM Na_2_GTP in 290 mOsm and pH 7.3 adjusted by KOH. HEKA EPC-10 amplifier double (HEKA Elektronik) was applied. The slice image was monitored under an upright Eclipse FN1 microscope (Nikon) through the infrared ray difference interference contrast (IR-DIC) optics with 400X magnification. An excitatory postsynaptic current (EPSC) was recorded in the voltage clamp mode at –70 mV holding potential in a CA1 pyramidal neuron. The access resistance in the recording cell was below 40 MΩ with marginal 20% tolerance. To stimulate the neuron, a bipolar electrode (FHC) in the external stimulator Iso-flex (A.M.P.I.) was positioned at Schaffer collateral at a distance of 200~400 μm from the recording electrode. Test stimulation pulses were applied at the same site every 30 s with 30–40% intensities from max EPSC amplitude 3 min before the following theta burst stimulation (TBS) for LTP induction. TBS consisted of four trains with 10-sec intervals, and each train was composed of 10 bursts at 5 Hz, with each burst having four pulses at 100 Hz. EPSCs were recorded for 20 min after TBS application. Data were filtered at 1 kHz and analyzed with Clampfit software (Molecular Devices).

### Immunohistochemical analysis

Mice were perfused with PBS and followed by 4% paraformaldehyde. Brain hemispheres were embedded sagittally in paraffin and prepared into sections of 5 μm slices [32]. Sections were deparaffinized, and antigen retrieval was performed in 10 mM sodium citrate buffer (pH 6.0) (Sigma-Aldrich, S4641). The sections were incubated with primary antibodies, followed by secondary antibodies and counterstained with DAPI. The immunofluorescent images were captured using an LSM700 microscope (Carl Zeiss). Images were quantified with the Icy (Quantitative Image Analysis Unit) and Zen (Carl Zeiss) softwares. Cell counting was performed by investigators who are blind to treatment condition and randomly allocated to groups.

### Aβ ELISA

The cerebral cortex was homogenized in buffer (250 mM Sucrose, 20 mM Tris HCl, pH 7.4, 1 mM EDGA, 1 mM EGTA, protease inhibitors). Homogenized samples were mixed with cold formic acid and sonicated for 1 min. After centrifugation at 135,000 x *g* for 1 h at 4 °C, supernatants were diluted into formic acid neutralization solution (1 M Tris HCl, 0.5 M Na_2_HPO_4_, 0.05% NaN_3_). Concentration of insoluble Aβ42 and Aβ40 levels and plasma Aβ40 levels were determined by sandwich ELISA kit (Invitrogen, KHB3441/KHB3481).

### Cell cultures

Primary neurons were derived from day 17–18 ICR mice embryo. Cells were cultured in neurobasal medium supplemented with 2% B27, 100 units/ml penicillin, 100 μg/ml streptomycin, and 2 mM L-glutamine. SH-SY5Y neuroblastoma cells were cultured in DMEM/F12 nutrient mixture (Invitrogen) supplemented with 10% fetal bovine serum, 100 units/ml penicillin and 100 μg/ml streptomycin. The shRNAs [control (Santa Cruz, sc-108060), TFEB (Santa Cruz, sc-38510-SH), MEK1 (Dharmacon, V3SM11241-234594572), and MEK2 (Dharmacon, V3SM11241-235104473)] were transfected using lipofectamine (Thermo, 11668019).

### Aβ42 oligomer preparation

Human Aβ42 peptide (Anaspec, AS-64129-1, 1 mg) was dissolved in 100 μl of DMSO by vortexing for 30 min at room temperature and added to 900 μl of PBS. It was incubated at 4 °C for 24 h for oligomer formation as described previously [33].

### Immunocytochemistry

Cells were washed with PBS, fixed in 4% paraformaldehyde for 15 min and permeabilized in 0.1% Triton X-100 for 5 min. Cells were placed in blocking solution (5% BSA) for 1 hr and incubated with primary antibodies for 2 h at room temperature. After washing, cells were incubated with secondary antibodies overnight. Coverslips were mounted using mounting medium (Biomeda). The coefficient percentage was calculated using the pixels above the threshold of fluorescence intensities. For measuring intra-lysosomal pH, cells were incubated with 500 nM LysoTracker Red DND-99 (Invitrogen) for 30 min at 37 °C following the manufacturer’s instructions. The fluorescence intensity was observed under confocal microscopy using an LSM700 microscope (Carl Zeiss). The number of LysoTracker puncta was analyzed with the Icy software (Quantitative Image Analysis Unit).

### Dendritic spine measurement

Primary hippocampal neurons were transfected with GFP plasmid DNA using lipofectamine (Invitrogen, 11668019) and the fluorescence intensity was observed under confocal microscopy using an LSM880 microscope (Carl Zeiss).

### Protein extraction and Western blotting

Proteins were extracted with RIPA buffer (65 mM Tris-base, 150 mM NaCl, 1% NP-40, 0.25% Na deoxycholate, 1 mM EDTA, protease inhibitors, pH 7.4) and centrifuged at 13,000 rpm for 20 min at 4 °C. For subcellular fractionation, cells were lysed with buffer (250 mM Sucrose, 20 mM HEPES, pH 7.4, 10 mM KCl, 1.5 mM MgCl_2_, 1 mM EGTA, 1 mM EDTA, 1 mM DTT, protease inhibitor) for 30 min on ice, followed by centrifugation at 720 *g* for 5 min at 4 °C. Supernatants were centrifuged at 15,000 rpm for 10 min at 4 °C, and the supernatant was used as the cytosol fraction. After centrifugation at 720 *g* for 5 min, pellets were washed with lysis buffer and dissolved in nuclear lysis buffer (50 mM Tris HCl, pH 8.0, 150 mM NaCl, 1% NP40, 0.5% sodium deoxycholate, 0.1% SDS, 10% glycerol) as the nuclear fraction. AD patient and age-matched control whole brain lysates were purchased from Genetex, MyBioSource, and BioChain (Supplementary Table 1). Protein samples were subjected to SDS-PAGE and transferred to nitrocellulose or polyvinylidene fluoride membrane. The membranes were blocked with 5% nonfat milk in TBS with 0.1% Tween 20 for 1 h at room temperature, then incubated with primary antibodies overnight at 4 °C. After washing, membranes were incubated with secondary antibodies for 2 h at room temperature. Peroxidase activity was visualized with enhanced chemiluminescence and quantified using a LAS-4000 system (Fuji Film). Antibodies used in this study were listed in Supplementary Table 2.

### Cathepsin B activity assay

Cathepsin B activity was measured from cell lysate using cathepsin B activity kit (Calbiochem, CBA001) according to the supplier’s instruction. Briefly, the lysates were incubated on ice for 30 min and centrifuged at 13,000 rpm for 20 min. Cathepsin B activity was then measured from supernatant using synthetic substrate at an excitation wavelength of 360 nm and an emission wavelength of 460 nm.

### TUNEL and Lactate dehydrogenase (LDH) assay

DNA fragmentation was determined using DeadEnd™ Fluorometric TUNEL System (Promega, G3250) according to the supplier’s instruction. Briefly, cells were fixed, permeabilized, labeled with the TUNEL reagent and fluorescent dUTP mixture for 1 h at 37 °C. The fluorescence intensity was observed under confocal microscopy using an LSM880 microscope (Carl Zeiss). The LDH activity in the was measured by using LDH Cytotoxicity Detection Kit (Roche, 04744934001) according to the manufacturer’s instructions.

### Quantitative real-time PCR (qRT-PCR)

Total RNA was extracted using TRIzol (Invitrogen, 15596018). Reverse transcription was performed using M-MLV reverse transcriptase (Invitrogen, 28025013). qRT-PCR was performed using the SYBR^TM^ Green PCR master mix (Thermo, 4367659) according to the manufacturer’s guidelines in QuantStudio 3 (Invitrogen, A28567). Results were expressed relative to the housekeeping gene GAPDH. The primer sets are indicated in Supplementary Table 3.

### Statistical analysis

All data were analyzed using GraphPad Prism 9 software. Statistical significance was assessed via Student’s t-test, one-way ANOVA or two-way ANOVA test followed by Dunnett’s post hoc analysis. Assessments with *p* < 0.05 were considered significant.

## Results

### Trametinib improves the cognitive deficits in the 5XFAD model

**Fig. 1:**
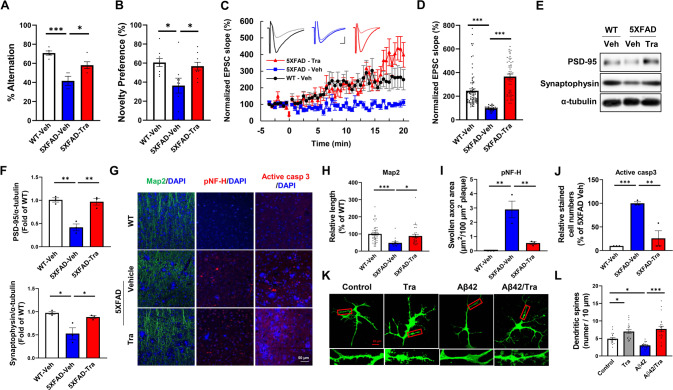
Trametinib rescues cognitive deficits and synaptic function in 5XFAD mice. **A** The 5-months 5XFAD transgenic mice were administered vehicle or 0.1 mg/kg of trametinib for 10 weeks by oral gavage once a day. At the end of the administration, Y-maze test was performed and the average ratio for the alternation in 3 min was calculated. Data were presented as the mean ± S.E.M. One-way ANOVA followed by Dunnett’s post hoc analysis (*F*_(2, 11)_ = 16.5, *p* = 0.0005; *n* = 4–5). **B** Novel object recognition test was performed and the average ratio of the time of investigations in 10 min was calculated. Data were presented as the mean ± S.E.M. One-way ANOVA followed by Dunnett’s post hoc analysis (*F*_(2, 24)_ = 5.335, *p* = 0.0121; *n* = 9). **C** Normalized EPSC slope in LTP recordings from the CA1 recording electrode. The baseline was stabilized for 3 min before TBS induction (red arrow) and the following 20 min recording in WT-vehicle (black circle; *n* = 8 slices from 6 mice), 5XFAD-vehicle (blue square; *n* = 4 slices from 3 mice), and 5XFAD-trametinib (red triangle; n = 6 slices from 3 mice). Representative EPSCs are displayed for each type with baseline (pale color) and response at 20 min (vivid color). Scalebar: 20 ms, 100pA. **D** Average of normalized EPSC slopes from 15.5 min to 20 min. Data were presented as the mean ± S.E.M. One-way ANOVA followed by Dunnett’s post hoc analysis (*F*_(2, 153)_ = 36.08, *p* < 0.001). **E** Representative western blot analysis of 5XFAD mice brain cortex lysates for PSD-95, and synaptophysin. α-tubulin was used as loading control. **F** Bars correspond to densitometric analysis of levels of PSD-95 (*F*_(2, 6)_ = 25.77, *p* = 0.0011; *n* = 3) and synaptophysin (*F*_(2, 6)_ = 9.836, *p* = 0.0128; *n* = 3). Normalized to the WT-vehicle group. Data were presented as the mean ± S.E.M. One-way ANOVA followed by Dunnett’s post hoc analysis. **G** Immunofluorescence staining images of MAP2, pNF-H, active caspase 3 in the cortex layer V of 5XFAD mice. Scale bars, 50 μm. **H**-**J** Quantification of neurite length (*F*_(2, 98)_ = 7.385, *p* = 0.001) (**H**), pNF-H-positive bulb-like swollen axon area (*F*_(2, 6)_ = 20.57, *p* = 0.0021) (**I**), and number of apoptotic cells (*F*_(2, 6)_ = 25.73, *p* = 0.0011) (**J**) in the cortex layer V (*n* = 3 sagittal sections from each mouse, *n* = 3 mice per group). Normalized to the WT-vehicle group. Data were presented as the mean ± S.E.M. One-way ANOVA followed by Dunnett’s post hoc analysis. **K** Primary hippocampal neurons (DIV22) were transfected with GFP plasmid DNA, treated with 100 nM trametinib and/or 5 μM Aβ42 oligomer for 48 h, and dendritic spine density were measured. **L** Quantification of number of dendritic spines. Scale bar = 20 μm. Data were presented as the mean ± S.E.M. Two-way ANOVA followed by Dunnett’s post hoc analysis (*F*_(3, 55)_ = 20.12, *p* < 0.001; *n* = 17). *p < 0.05; **p < 0.01; ***p < 0.001.

For assessment of trametinib’s brain access, we first tested blood-brain barrier (BBB) penetration in mice. After a single oral administration to ICR mice, trametinib penetrated the BBB, and the brain to plasma exposure ratio (AUC ratio) was in the range of 47.7% to 64.2% depending on trametinib doses (Supplementary Fig. 1A, Supplementary Table 4). After daily administration of trametinib for 1-4 weeks, the level of phosphorylated ERK1/2 in the brain clearly decreased (Supplementary Fig. 1B). To determine the effect of trametinib in AD-related pathology, 5-month-old 5XFAD mice were administered either vehicle or 0.1 mg/kg of trametinib for 10 weeks by oral gavage once a day. In the Y-maze test used to assess short-term spatial working memory, we observed a significant decline in spontaneous alteration in vehicle-administered 5XFAD mice compared to WT mice. In contrast, trametinib-treated 5XFAD mice showed improved spatial working memory compared to non-treated 5XFAD (Fig. 1A, Supplementary Fig. 2A). The total arm entries were not significantly different among groups in the Y-maze test (Supplementary Fig. 2B). We further tested nonspatial long-term memory function using the novel object recognition test. Trametinib also improved novel object recognition memory of 5XFAD mice compared to vehicle-treated 5XFAD (Fig. 1B). We did not observe any significant difference between vehicle-administered and trametinib-treated WT mice in Y-maze and novel object recognition tests (Supplementary Fig. 2C, D). These results demonstrate that orally administered trametinib improves cognitive dysfunctions in 5XFAD.

### Trametinib recovers synaptic and network dysfunction in the 5XFAD mouse brain

The EPSCs were recorded at the hippocampal CA1 region to compare the LTP between wild-type and 5XFAD mice at the age of 8 months. As reported previously [34], LTP in the 5XFAD-vehicle group was significantly impaired compared with vehicle-treated WT mice (Fig. 1C, D). Administration of trametinib for three months restored LTP of 5XFAD mice to WT levels. We next analyzed the levels of presynaptic marker synaptophysin and postsynaptic marker PSD-95 to determine the protective effect of trametinib against synaptic degeneration in the cortex of 5XFAD mouse [35]. We found that synaptophysin and PSD-95 levels were decreased in the cortex of 5XFAD and trametinib recovered them to WT mice levels (Fig. 1E, F). These observations indicated that trametinib recovers the impaired neuronal networks in the cortex of 5XFAD mice. Similar to the observed mouse brain data, trametinib recovered Aβ42-induced reduction of synaptophysin and PSD-95 in primary cultured neurons as assessed by immunoblot (Supplementary Fig. 3A–C) and immunocytochemistry (Supplementary Fig. 3D, E).

To determine if trametinib recovers the malformed dendrites in the 5XFAD mouse cortex, we measured the length of dendrites using the dendritic marker MAP2. While the length of dendrites in the cortex of 5XFAD mice was significantly shortened, it was recovered in the trametinib-treated 5XFAD mice (Fig. 1G, H). In addition, pNF-H-positive bulb-like swollen axons, an indicator of axonal deterioration due to Aβ plaque accumulation in the brains of AD patients and aged monkeys [36, 37], were increased in vehicle-administered 5XFAD compared to those in WT, while it was significantly reduced by trametinib in 5XFAD mice (Fig. 1G, I). The increase of synaptic protein levels induces spine formation [38, 39]. Consistently, we found that Aβ42 oligomers significantly decreased the number of dendritic spines, while trametinib restored them in primary hippocampal neurons (Fig. 1K, L).

The 5XFAD mice showed active caspase-3-positive apoptotic neuronal death caused by amyloid burden [40], while trametinib treatment reduced active caspase-3 in the cortex of 5XFAD mice (Fig. 1G, J). To further clarify the neuroprotective effect of trametinib, we tested whether trametinib treatment protects SH-SY5Y cells and primary cortical neurons from apoptosis induced by Aβ42 oligomer. Trametinib reduced Aβ42 oligomer-induced cleavage of PARP and activation of caspase-3 in SH-SY5Y cells (Supplementary Fig. 4A–C). Moreover, trametinib prevented Aβ42 oligomer-induced death of primary cortical neurons as assessed by TUNEL (Supplementary Fig. 4D, E) or LDH assay (Supplementary Fig. 4F). These results imply that trametinib not only protects synaptic integrity but also protects axons and dendrites against amyloid plaque toxicity.

### Trametinib reduces Aβ deposition in 5XFAD mice

**Fig. 2:**
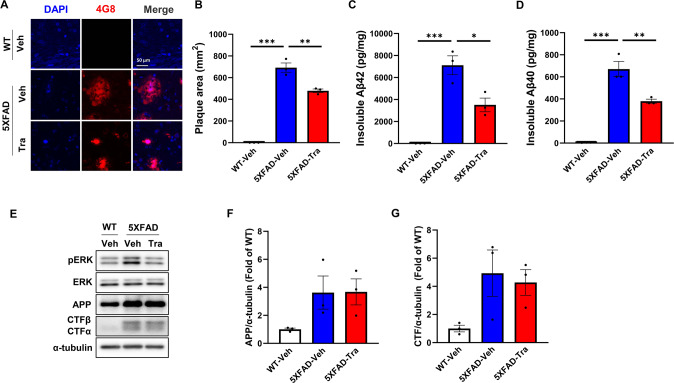
Trametinib decreases amyloid β deposition in 5XFAD mice. **A** Representative images of cortex layer V immunostained with the Aβ antibody (clone 4G8) (*n* = 3 sagittal sections from each mouse, *n* = 3 mice per group). Scale bars, 50 μm. **B** Quantification of plaque area. Data were presented as the mean ± S.E.M. One-way ANOVA followed by Dunnett’s post hoc analysis (*F*_(2, 6)_ = 178.1, *p* < 0.001). **C**, **D** The levels of insoluble Aβ42 (*F*_(2, 6)_ = 34.07, *p* = 0.0005) (**C**) and Aβ40 (*F*_(2, 6)_ = 66.11, *p* < 0.001) (**D**) in cortex of 5XFAD mice were measured by ELISA. Data were presented as the mean ± S.E.M. One-way ANOVA followed by Dunnett’s post hoc analysis. **E** Representative western blot analysis of 5XFAD mice brain cortex lysates for indicated proteins. α-tubulin was used as loading control. **F** Bars correspond to densitometric analysis of level of APP. Data were presented as the mean ± S.E.M. One-way ANOVA followed by Dunnett’s post hoc analysis (*F*_(2, 6)_ = 3.095, *p* = 0.1193; *n* = 3). **G** Bars correspond to densitometric analysis of level of CTF (*F*_(2, 6)_ = 3.673, *p* = 0.0909; *n* = 3). Data were presented as the mean ± S.E.M. One-way ANOVA followed by Dunnett’s post hoc analysis. *p < 0.05; **p < 0.01; ***p < 0.001 versus 5XFAD-Veh group.

When we compared Aβ deposition in the cortex of 5XFAD mice, trametinib administration significantly reduced the area of Aβ plaques (Fig. 2A, B). We then analyzed levels of insoluble amyloids in the cortex of 5XFAD by using ELISA. The levels of insoluble Aβ42 and Aβ40 in trametinib-administered 5XFAD were reduced by 51% and 49%, respectively, compared with those in the vehicle-administered 5XFAD mice (Fig. 2C, D). The level of Aβ is determined by its rate of production and degradation [41, 42]. To ask whether trametinib affects Aβ production, we determined the amyloid precursor protein (APP) levels and its intermediate cleavage products, C-terminal fragments (CTFs). Trametinib treatment did not change the levels of APP, CTFβ, and CTFα in the cortex of 5XFAD mice (Fig. 2E–G). In addition, the levels of MMP-9 and neprilysin, Aβ degrading proteases [41] were not changed by trametinib in 5XFAD mice and Aβ-treated primary cortical neurons (Supplementary Fig. 5A–F). Moreover, there is no difference in the plasma level of Aβ40 between vehicle- and trametinib-treated 5XFAD mice, suggesting that trametinib does not affect the efflux of Aβ from the brain (Supplementary Fig. 5G). Taken together, these data suggest that trametinib reduces Aβ deposition probably by enhancing intracellular degradation of Aβ.

### Trametinib enhances autophagic lysosomal activity in 5XFAD mice

**Fig. 3:**
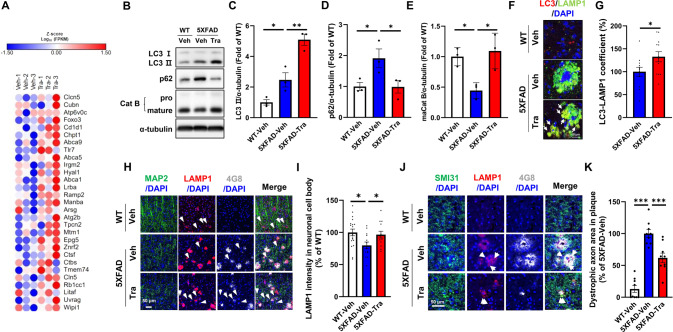
Trametinib increases autophagic lysosomal activity in 5XFAD mice. **A** Analysis of the brain lysates from normal mice administered with trametinib for 2 weeks. Heatmap of RNA-Seq for 29 selected genes from autophagic lysosomal genes. The heatmap of RNA-Seq shows log10 FPKM (fragments per kilobase per million reads mapped) values for 29 selected genes (rows) and 6 samples (vehicle or trametinib administered, *n* = 3 in each group). Color corresponds to per-gene z-score that is calculated from log10 FPKM and represented using Morpheus heatmap program. **B** Representative western blot analysis of 5XFAD mice brain cortex lysates for indicated proteins. **C** Bars correspond to densitometric analysis of LC3II/α-tubulin. Data were presented as the mean ± S.E.M. One-way ANOVA followed by Dunnett’s post hoc analysis (*F*_(2, 6)_ = 33.8, *p* = 0.0005; *n* = 3). **D** Bars correspond to densitometric analysis of p62. Data were presented as the mean ± S.E.M. One-way ANOVA followed by Dunnett’s post hoc analysis (*F*_(2, 6)_ = 5.603, *p* = 0.0424; *n* = 3). **E** Bars correspond to densitometric analysis of cathepsin B. Data were presented as the mean ± S.E.M. One-way ANOVA followed by Dunnett’s post hoc analysis (*F*_(2, 6)_ =  8.872, *p* = 0.0161; *n* = 3). **F** Immunofluorescence images of LC3, and LAMP1 in cortex layer V. Arrows indicate co-stained regions (*n* = 3 sagittal sections from each mouse, *n* = 3 mice per group). Scale bars, 10 μm. **G** Quantification of the co-stained ratio with LC3 and LAMP1. Data were presented as the mean ± S.E.M. Student’s *t*-test (*p* = 0.0381). **H**-**K** The 9-month-old 5XFAD mice were administered either vehicle or 0.1 mg/kg of trametinib for 6 weeks by oral gavage once a day. Immunofluorescence staining images of Aβ, LAMP1, and MAP2 in the cortex of 5XFAD mice (**H)**. Scale bars, 50 μm. Quantification of LAMP1 intensity within cell body of MAP2 + neurons (**I)** (*F*_(2, 38)_ = 4.320, *p* = 0.0204). Immunofluorescence staining images of Aβ, LAMP1, and SMI31 in the cortex of 5XFAD mice (**J**). Scale bars, 50  μm. Quantification of the area of dystrophic axon within plaque (**K)** (*F*_(2, 24)_ = 46.67, *p* < 0.0001). Data were presented as the mean ± S.E.M. One-way ANOVA followed by Dunnett’s post hoc analysis. *p < 0.05; **p < 0.01; ***p < 0.001 versus 5XFAD-Veh group.

To investigate how trametinib regulates synaptic dysfunction and Aβ deposition, we identified genes transcriptionally regulated by trametinib in bulk RNA-Seq from whole brains of wild-type C57BL/6 mice. A total of upregulated 836 differentially expressed genes (DEGs) and downregulated 575 DEGs were identified between the vehicle- and trametinib-treated groups (Supplementary Table 5, 6). Gene ontology analysis revealed that trametinib upregulated the negative regulation of transcription by RNA polymerase and autophagy-related gene expression, but downregulated sensory perception of the chemical stimulus, positive regulation of neuron death and negative regulation of amyloid-beta clearance-related genes (Supplementary Fig. 6). Given that trametinib increases Aβ degradation in 5XFAD mice and regulates autophagy-related gene expression from RNA-Seq analysis and that the autophagic lysosomal pathway is an important degradation mechanism in amyloid pathology [43], we further examined the expression patterns of the previously reported 322 autophagic lysosomal related genes [44]. Whole RNA-Seq analysis revealed that trametinib upregulated the expression of a subset of autophagic lysosomal associated genes. Specifically, 29 genes that function within the autophagic pathway were differentially upregulated as illustrated by the gene expression heatmap (Fig. 3A, Supplementary Table 7). These results signify that trametinib induces autophagic lysosomal activity in the brain. Indeed, the level of autophagic marker LC3-II, which was higher in the 5XFAD mice than in WT mice, further increased in the trametinib-administered 5XFAD mice (Fig. 3B, C, Supplementary Fig. 7A). In addition, the level of p62, a marker of autophagic flux [45–47], increased in the 5XFAD mice compared to WT mice, while it was reduced by trametinib in 5XFAD mice (Fig. 3B, D, Supplementary Fig. 7A). While the level of mature cathepsin B, a lysosomal protease, decreased in the vehicle-administered 5XFAD mice, trametinib increased its level in 5XFAD mice (Fig. 3B, E). Furthermore, increased LC3-LAMP1 colocalization (Fig. 3F, G) in the cortex indicates that trametinib induces autophagosome-lysosome fusion. These results indicate that trametinib administration rescued the impaired autophagosome-lysosome fusion and lysosomal maturation seen in the 5XFAD mice.

Immature lysosomes within swollen neuronal axons accumulate at amyloid plaques in 5XFAD mice, and this accumulation prevents lysosomal transport to the cell body where mature lysosomes fuse with autophagosomes to acquire degradative activity [11, 48, 49]. Consistently, mature lysosomes in neuronal cell body surrounding plaques as determined by immunofluorescence staining with MAP2, LAMP1 and Aβ were decreased, while axonal lysosomes co-stained with LAMP1 and SMI31, a neurofilament marker, were increased in vehicle-administered 5XFAD mice (Fig. 3H–K). Trametinib administration restored the reduction of mature lysosomes in the neuronal cell body and the induction of lysosomes in dystrophic axons in 5XFAD mice. Microglia cluster around the amyloid plaques and activate the inflammatory response in the AD brain [50, 51]. Since microglia are capable of clearing Aβ deposition through phagocytosis and degradation [52], we assessed whether the microglia contribute to lysosomal activity and Aβ uptake upon trametinib administration. While trametinib decreased the number and area of IBA1^+^ microglia in 5XFAD mice, it did not change the area ratio of Aβ within the IBA1^+^ microglia area and the intensity of LAMP1 in microglia (Supplementary Fig 8). Taken together, these results suggest that trametinib enhances the autophagic lysosomal activity primarily in neurons of 5XFAD mice.

### Trametinib increases autophagic flux to prevent Aβ-induced neurotoxicity

**Fig. 4:**
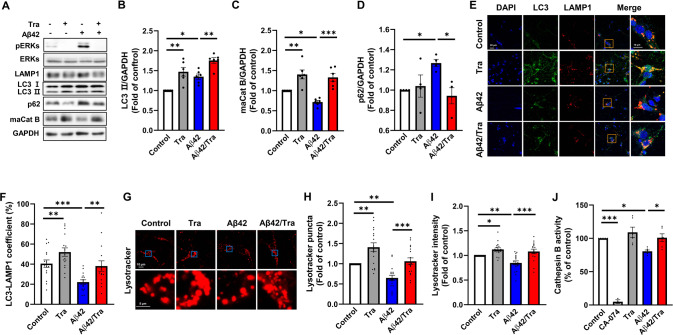
Trametinib increases autophagic flux in primary cortical neuron. **A** Representative western blot analysis of pERKs, ERKs, LAMP1, LC3, p62, and cathepsin B from cell lysates of primary cortical neuron. GAPDH was used as loading control. **B** Bars correspond to densitometric analysis of level of LC3II/ GAPDH. Data were presented as the mean ± S.E.M. Two-way ANOVA followed by Dunnett’s post hoc analysis (*F*_(3, 15)_ = 15.93, *p* < 0.0001; *n* = 5). **C** Bars correspond to densitometric analysis of level of cathepsin B. Data were presented as the mean ± S.E.M. Two-way ANOVA followed by Dunnett’s post hoc analysis (*F*_(3, 15)_ = 17.92, *p* < 0.0001; *n* = 5). **D** Bars correspond to densitometric analysis of level of p62. Data were presented as the mean ± S.E.M. Two-way ANOVA followed by Dunnett’s post hoc analysis (*F*_(3, 9)_ = 4.848, *p* = 0.0283; *n* = 4). **E** Quantification of the co-stained ratio with LC3 and LAMP1. Scale bars, 20 μm or 10 μm. **F** Quantification of the co-stained ratio with LC3 and LAMP1. Data were presented as the mean ± S.E.M. Two-way ANOVA followed by Dunnett’s post hoc analysis (*F*_(3, 44)_ = 10.18, *p* < 0.0001; *n* = 16). **G** Images of lysotracker staining in primary cortical neuron. Scale bars, 10 μm or 5 μm. **H** Quantification of number of lysotracker puncta of cells. Data were presented as the mean ± S.E.M. Two-way ANOVA followed by Dunnett’s post hoc analysis (*F*_(3, 48)_ = 17.1, *p* < 0.0001; *n* = 17). **I** Quantification of intensity of lysotracker of cells. Data were presented as the mean ± S.E.M. Two-way ANOVA followed by Dunnett’s post hoc analysis (*F*_(3, 48)_ = 12.99, *p* < 0.0001; *n* = 17). **J** Enzymatic activity of cathepsin B in primary cortical neuron was evaluated in three independent experiments. CA-074, cathepsin B inhibitor, was used as a negative control. Data were presented as the mean ± S.E.M. Two-way ANOVA followed by Dunnett’s post hoc analysis (*F*_(4, 17)_ = 55.28, *p* < 0.0001). *p < 0.05; **p < 0.01; ***p < 0.001.

We then asked whether trametinib upregulates the autophagic lysosomal pathway either by activating the autophagic activity or by enhancing autophagic flux [53]. LC3-II and pERKs levels increased upon Aβ oligomers treatment, while trametinib-mediated ERK inhibition further increased the level of LC3-II in primary cortical neurons (Fig. 4A, B). Trametinib also increased the level of mature cathepsin B despite the presence of Aβ42 oligomers. Trametinib had no effect on the LAMP1 level (Fig. 4A, C). Degradation of p62 was observed with trametinib treatment in the presence of Aβ42 oligomers (Fig. 4A, D). Trametinib-mediated induction of LC3-II and mature cathepsin B and degradation of p62 was also observed in SH-SY5Y cells in the presence of Aβ42 oligomers (Supplementary Fig. 7B–D). The role of trametinib in autophagosome-lysosome fusion was further confirmed using bafilomycin A1 which interrupts the fusion of autophagosomes and lysosomes by inhibiting V-ATPase [54–56]. Treatment with bafilomycin A1 eliminated the effect of trametinib on increasing mature cathepsin B and decreasing p62 in the presence of Aβ42 oligomers (Supplementary Fig. 7E). Moreover, trametinib increased the co-staining of LC3-LAMP1 even in the presence of Aβ42 oligomers in primary cortical neurons and SH-SY5Y cells (Fig. 4E, F, Supplementary 7F, G). To assess lysosomal acidification, we measured lysotracker-positive acidic puncta and intensity. Trametinib treatment resulted in increased lysotracker-positive acidic puncta and intensity in the presence of Aβ42 oligomers in both primary cortical neurons and SH-SY5Y cells (Fig. 4G–I, Supplementary Fig. 7F, H, I), suggesting that trametinib elicits activation of lysosome function. The enzymatic assay revealed that trametinib increases cathepsin B activity in the presence of Aβ42 oligomers (Fig. 4J). Trametinib-mediated recovery of Aβ42-induced dendritic spine loss was prevented by lysosome and protease inhibitors, NH_4_Cl, and leupeptin in primary hippocampal neurons (Supplementary Fig. 9). Taken together, these results imply that trametinib leads to an upregulation of the autophagic lysosomal pathway by enhancing autophagic flux, supporting its role as a key degradation mechanism in amyloid pathology.

### Trametinib promotes the autophagic lysosomal pathway through TFEB

**Fig. 5:**
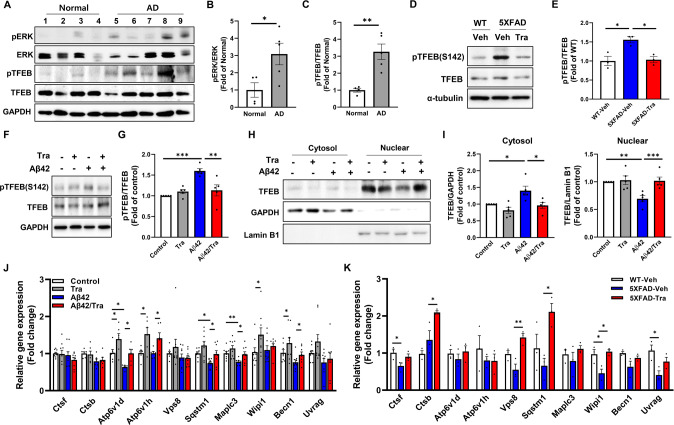
Trametinib regulates the expression of TFEB-regulated autophagy-lysosome genes. **A** Representative western blot analysis of Human brain lysates for indicated proteins. **B** Bars correspond to densitometric analysis of level of pERK/ERK. Data were presented as the mean ±  S.E.M. Student’s *t*-test (*p* = 0.0349; *n* = 4–5). **C** Bars correspond to densitometric analysis of level of pTFEB/TFEB. Data were presented as the mean ± S.E.M. Student’s *t*-test (*p* = 0.0036; *n* = 4–5). **D** Representative western blot analysis of 5XFAD mice brain cortex lysates for indicated proteins. **E** Bars correspond to densitometric analysis of pTFEB/TFEB. Data were presented as the mean ± S.E.M. One-way ANOVA followed by Dunnett’s post hoc analysis (*F*_(2, 6)_  = 10.57, *p* = 0.0108; *n* = 3). **F** Representative western blot analysis of pTFEB(S142) and TFEB from cell lysates of primary cortical neuron. GAPDH was used as loading control. **G** Bars correspond to densitometric analysis of level of pTFEB(S142). Data were presented as the mean ± S.E.M. Two-way ANOVA followed by Dunnett’s post hoc analysis (*F*_(3, 12)_ = 15.49, *p* = 0.0002; *n* = 5). **H** Representative western blot analysis of TFEB in the cytosolic and nuclear fractions of primary cortical neuron. GAPDH and lamin B1 were used as cytosolic and nuclear fractions marker, respectively. **I** Bars correspond to densitometric analysis of level of TFEB in the cytosolic (*F*_(3, 12)_ = 7.834, *p* = 0.0037; *n* = 5) and nuclear fractions (*F*_(3, 12)_ = 13.12, *p* = 0.0004; *n* = 5). Data were presented as the mean ± S.E.M. Two-way ANOVA followed by Dunnett’s post hoc analysis. **J** qPCR of TFEB target genes in primary cortical neuron (*F*_(3, 24)_ = 1.234, *p* = 0.3191, Ctsf; *F*_(3, 15)_ = 2.644, *p* = 0.0871, Ctsb; *F*_(3, 15)_ = 12.61, *p* = 0.0002, Atp6v1d; *F*_(3, 15)_ = 6.137, *p* = 0.0062, Atp6v1h; *F*_(3, 24)_ = 1.107, *p* = 0.365, Vps8; *F*_(3, 24)_ = 10.33, *p* = 0.0001, Sqstm1; *F*_(3, 24)_ = 10.90, *p* = 0.0001, Maplc3; *F*_(3, 24)_ = 3.08, *p* = 0.0466, Wipi1; *F*_(3, 22)_ = 9.804, *p* = 0.0003, Becn1; *F*_(3, 24)_ = 3.536, *p* = 0.0291, Uvrag; *n* = 6–9). Data were presented as the mean ± S.E.M. Two-way ANOVA followed by Dunnett’s post hoc analysis. **K** Expression analysis of the TFEB target genes by qPCR in the cortex of 5XFAD (*F*_(2, 6)_ = 4.678, *p* = 0.0596, Ctsf; *F*_(2, 6)_ = 13.98, *p* = 0.0056, Ctsb; *F*_(2, 6)_ = 0.6408, *p* = 0.5595, Atp6v1d; *F*_(2, 6)_ = 0.6372, *p* = 0.5611, Atp6v1h; *F*_(2, 6)_ = 13, *p* = 0.0066, Vps8; *F*_(2, 6)_ = 6.976, *p* = 0.0272, Sqstm1; *F*_(2, 6)_ = 1.042, *p* = 0.4089, Maplc3; *F*_(2, 6)_ = 9.417, *p* = 0.0141, Wipi1; *F*_(2, 6)_ = 3.874, *p* = 0.0831, Becn1; *F*_(2, 6)_ = 6.938, *p* = 0.0275, Uvrag; *n* = 3). Data were presented as the mean ± S.E.M. One-way ANOVA followed by Dunnett’s post hoc analysis. *p < 0.05; **p < 0.01; ***p < 0.001.

The autophagic lysosomal pathway is shown to be transcriptionally regulated by TFEB, of which nuclear localization and transcription factor activity are inhibited through phosphorylation at Ser142 by ERK [27, 57]. We first examined the activation of the ERK-TFEB axis in AD patient brains. The levels of pTFEB and pERK are higher in AD patient brains than those in age-matched normal brains (Fig. 5A–C, Supplementary Table 1). In addition, we observed increased pERK within neurons and pTFEB in the brains of 5XFAD mice compared to WT mice, while they were reduced by trametinib administration (Fig. 5D, E, Supplementary Figs. 7A, and 10). We also found that trametinib reduced the Aβ42 oligomers-induced phosphorylation of TFEB in the primary cortical neuron (Fig. 5F, G). While the addition of Aβ oligomers reduced the nuclear level of TFEB, trametinib treatment restored the nuclear level of TFEB in primary cortical neurons and SH-SY5Y cells (Fig. 5H, I, Supplementary Fig. 11A). These data indicated that trametinib inhibited TFEB phosphorylation and localized TFEB to the nucleus resulting in upregulation of autophagic lysosomal genes as we observed in RNA-Seq analysis of trametinib-administered mouse brains (Fig. 3A). We then chose the known TFEB targets [27, 58] among upregulated autophagic lysosomal genes and confirmed their expression using qPCR from primary cortical neurons and 5XFAD cortex. Aβ42 oligomers reduced the expression of TFEB target genes, and trametinib treatment significantly increased the expression of autophagic-lysosomal genes in primary cortical neurons (Fig. 5J). The decreased expression of TFEB target genes seen in 5XFAD mice compared to WT mice was also upregulated by trametinib (Fig. 5K). We then determined the direct effect of TFEB regulated by trametinib on Aβ42-induced autophagic lysosomal dysregulation and cell death. Knockdown of TFEB eliminated the effect of trametinib on the increase of mature cathepsin B, degradation of p62, and inhibition of caspase-3 in primary cortical neurons treated with Aβ42 oligomers (Supplementary Fig. 11B–E), suggesting that TFEB is required for the action of trametinib.

## Discussion

The major findings of this paper are functional neuronal recovery in AD due to clinically available trametinib’s pharmacological inhibition of MEK. Trametinib administration ameliorated memory deficits and rectified synaptic dysfunction by improving LTP and increasing synaptic protein expression in 5XFAD mice. As a mechanism of action, trametinib enhanced TFEB-dependent autophagic lysosomal function, resulting in the decrease of Aβ accumulation in the cortex of 5XFAD mice. Thus, we first demonstrated MEK1/2-mediated regulation of TFEB-dependent autophagic lysosomal function by trametinib in the 5XFAD mouse model of amyloid deposition. We chose to use 5XFAD mice that display early and aggressive phenotypes of amyloid plaques and intraneuronal Aβ and are used in autophagy research [59–61]. Since there is also a close connection between tau pathology and auto-lysosomal dysfunction in AD [62, 63], further research is required if trametinib enhances the autophagic clearance of pathologic tau.

Recent studies have shown that inhibition of Ras/ERK signaling increases autophagic flux in cancer, a protective mechanism for cell survival [64, 65]. The autophagic lysosomal pathway is an essential mechanism to clear abnormal proteins involved in neurodegenerative disease pathology. Thus, eliminating aggregated proteins such as Aβ and tau proteins by enhancing autophagy is a potential therapeutic approach for AD treatment. While several drugs enhancing autophagy have been tested in AD clinical trials [66, 67], the link between MEK/ERK signaling and autophagic lysosomal processes has not been fully understood in AD. Our study showed that trametinib’s inhibition of MEK/ERK signaling is important for inducing autophagic lysosomal activity in AD. Indeed, we confirmed that other MEK inhibitors (AZD8330, Binimetinib, SL-327, Refametinib, PD318088, PD0325901, Ro5126766) [68–73] also increased autophagosome-lysosome fusion in the presence of Aβ42 oligomers (Supplementary Fig. 12) and prevented Aβ42 oligomer-induced cell death (Supplementary Fig. 13) similar to trametinib. Moreover, knockdown of MEK increased autophagic lysosomal activity and decreased pTFEB level in the presence of Aβ42 oligomers (Supplementary Fig. 14), further supporting that trametinib’s suppression of MEK activity drove autophagic lysosomal activation in AD.

We observed that trametinib provides a neuroprotective effect in in vivo and in vitro AD models. Aβ-induced neurotoxicity causes activation of the Ras/ERK signaling pathway resulting in caspase-3 activation, which induces apoptotic neuronal death [74–76]. MEK inhibition by trametinib attenuated caspase-3 activation and DNA fragmentation, indicating the inhibition of apoptosis. Trametinib partially but significantly attenuated Aβ42-induced LDH release. Since Aβ42 induces both necrosis and apoptosis [77, 78], trametinib may lead to partial protection from cell death by the Aβ42 oligomer. It has been known that Ras/ERK signaling regulates APP processing and Aβ generation. MEK inhibition upregulates γ-secretase activity, which results in the decrease of CTF and the increase of Aβ in vitro [79]. It has been also reported that MEK suppression strongly inhibits the α-secretase activity in vitro [80]. Although there have been conflicting reports on MEK’s effect on Aβ production, we confirmed that MEK inhibition by trametinib did not affect APP and CTF levels, extracellular degradation of Aβ, and efflux of Aβ from the brain. However, MEK inhibition significantly reduced Aβ level by increasing mature lysosomes in the neuronal cell body and the reducing of lysosomes in dystrophic axons surrounding plaques in 5XFAD mice. On the contrary, trametinib did not change the area ratio of Aβ within the IBA1^+^ microglia area and the intensity of LAMP1 in microglia, suggesting that trametinib does not affect microglial degradation of Aβ and lysosomal activity, rather mainly enhances the autophagic lysosomal activity in neuron. Interestingly, however, increased number and area of IBA1^+^ microglia in 5XFAD mice were recovered by trametinib administration. Since microglia become readily activated upon apoptosis induction and contribute to engulfment of dying neurons [81–85], trametinib may indirectly contribute to inhibition of microglial activation by preventing neuronal cell death in 5XFAD mice.

Our RNA-Seq analysis showed that autophagic lysosomal-related genes were elevated by trametinib administration, indicating a transcriptional regulatory mechanism. We identified TFEB as a mechanism of action of trametinib in AD models. TFEB is a master regulator of gene expression in different steps of the autophagy process: lysosome and autophagy biogenesis, autophagosome formation, and autophagosome-lysosome fusion [27, 58]. We found that trametinib upregulated autophagic lysosomal-related genes, including some of the TFEB-regulated genes. Several studies suggest that the function of TFEB is related to the activity of autophagic lysosomal degradation in AD. TFEB is highly expressed in glial cells [86], thus activation of TFEB in astrocytes induces Aβ clearance and attenuates amyloid plaque [87], and the microglial expression of TFEB facilitates fibrillar Aβ degradation through upregulation of lysosomal biogenesis in APP/PS1 mice [88]. In addition, TFEB overexpression rescues memory deficits in the P301S tauopathy mouse model [89]. Future studies are required to determine whether trametinib regulates glial TFEB in different AD models. On the other hand, TFEB is also known to be expressed and have a role in neurons [90–96]. Consistently, our results further verified that MEK inhibition by trametinib suppresses TFEB phosphorylation at Ser142 and increases its nuclear translocation, resulting in upregulation of the expression of TFEB-regulated autophagic lysosomal genes in murine primary cortical neurons and in the brains of 5XFAD mice. Moreover, suppression of TFEB expression in primary cortical neurons eliminates the neuroprotective effect of trametinib against Aβ42 oligomers. Although we did not rule out the possibility that protective effect of trametinib in AD is via other ERK-dependent mechanisms in non-neuronal cells, we clearly showed that MEK inhibition by trametinib provides neuronal protection from Aβ burden by increasing autophagic lysosomal activity through TFEB activation in models of AD. Thus, therapeutic utility of MEK inhibition may have broad neuroprotective properties in other neurodegenerative diseases such as AD-related dementia and Parkinson’s disease where autophagic lysosomal activity plays a role.

## Supplementary information

Supplementary Information

Supplementary Table 5

Supplementary Table 6
