# Supplementary material for: MEK1/2 inhibition rescues neurodegeneration by TFEB-mediated activation of autophagic lysosomal function in a model of Alzheimer’s Disease
Source: Mol Psychiatry. 2022 Aug 10;27(11):4770–80. doi: 10.1038/s41380-022-01713-5 (PMC9734062; doi:10.1038/s41380-022-01713-5)
Supplement: Supplementary file 1 — Supplementary Information [file 41380_2022_1713_MOESM1_ESM.docx]

**Supplementary Information**

**MEK1/2 inhibition rescues neurodegeneration by TFEB-mediated activation of autophagic lysosomal function in a model of Alzheimer’s Disease**

Yoon Sun Chun, Mi-Yeon Kim, Sun-Young Lee, Mi Jeong Kim, Tae-Joon Hong, Jae Kyong Jeon, Dulguun Ganbat , Hyoung Tae Kim, Sang Seong Kim, Tae-In Kam, Sungho Han

Correspondence to: Tae-In Kam, Ph.D. ([tkam1@jhmi.edu](mailto:tkam1@jhmi.edu)) and Sungho Han, Ph.D. ([Han@genuv.com](mailto:Han@genuv.com))

**This file includes:**

Figures S1 to S14

Table S1 to S7

References

**Supplementary Figure Legends**

**
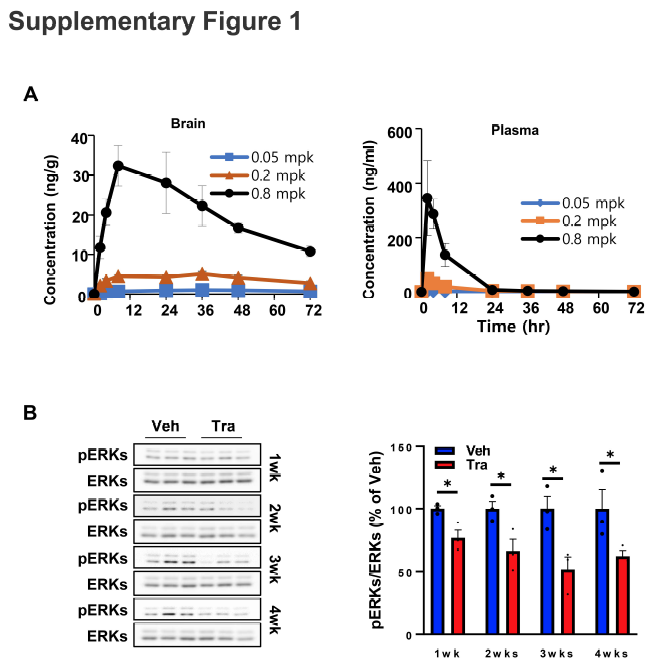
**

**Supplementary Figure 1. Trametinib penetration into the mouse brain.**

**(A)** Brain and plasma concentration-time profiles of trametinib after a single oral administration. **(B)** Representative western blot analysis of mice whole brain lysates for pERKs and ERKs. ERKs were included as a loading control. Bars correspond to densitometric analysis of level of pERK. Data were presented as the mean ± S.E.M. (*p*=0.0134, 1wk; *p*=0.0202, 2wks; *p*=0.013, 3wks; *p*=0.0388, 4wks; n = 3). *P* values were obtained by Student’s t-test. *p < 0.05 compared with the vehicle group.

**
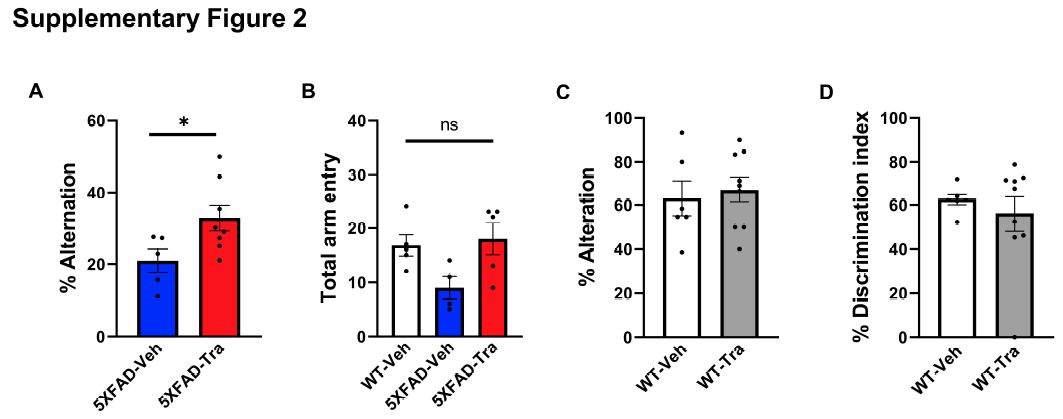
**

**Supplementary Figure 2. Trametinib restores cognitive deficits in 5XFAD mice.**

**(A)** The 7-months 5XFAD mice were administered vehicle or 0.1 mg/kg of trametinib for 1 months by oral gavage once a day. Y-maze test was performed and the average ratio for the alternation in 5 minutes was calculated (5XFAD-vehicle group; n = 5, 5XFAD-trametinib group; n = 8). *P* values were obtained by Student’s t-test (*p*=0.0419). **(B)** The 5-months 5XFAD mice were administered vehicle or 0.1 mg/kg of trametinib for 10 weeks by oral gavage once a day. At the end of the administration, behavioral tests were performed. Y-maze test was performed and the number of total arm entries in 3 minutes was counted. Data were presented as the mean ± S.E.M. One-way ANOVA followed by Dunnett’s post hoc analysis (*F*_(2, 6)_ = 3.703, *p*=0.0589). **(C, D)** The 5-months WT mice were administered vehicle or 0.1 mg/kg of trametinib for 10 weeks by oral gavage once a day (WT-vehicle group; n = 6, WT-trametinib group; n = 9). Y-maze test was performed and the average ratio for the alternation in 3 minutes was calculated **(C)** (*p*=0.6825). Novel object recognition test was performed and the average ratio of the number of investigations in 3 minutes was calculated **(D)** (*p*=0.5435). *P* values were obtained by Student’s t-test.

**
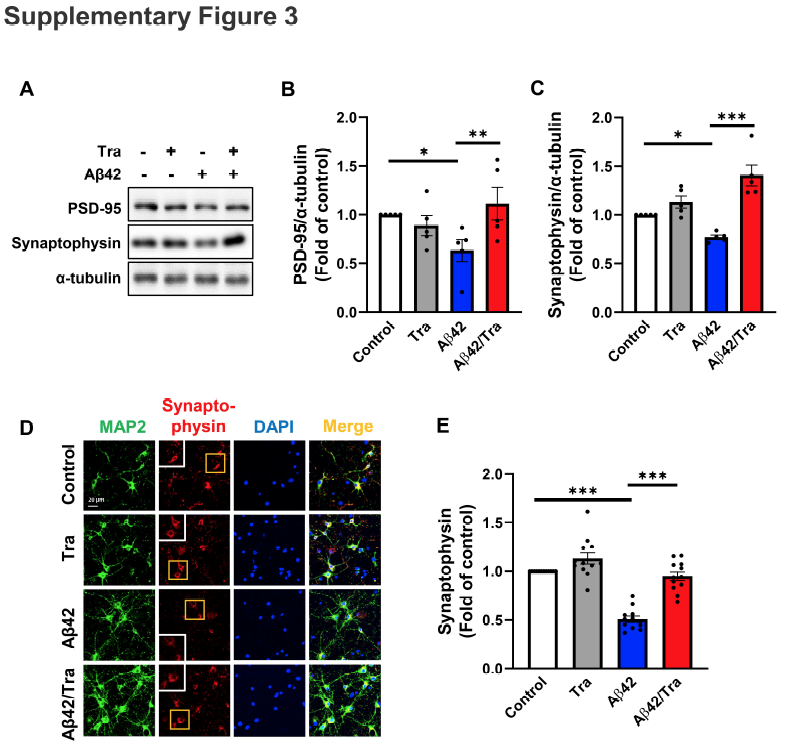
**

**Supplementary Figure 3. Trametinib rescues synaptic degeneration in primary cortical neuron.**

**(A)** Representative western blot analysis of PSD-95, and synaptophysin from cell lysates of primary cortical neuron. α-tubulin was used as loading control. **(B)** Bars correspond to densitometric analysis of level of PSD-95/α-tubulin. Data were presented as the mean ± S.E.M. Two-way ANOVA followed by Dunnett’s post hoc analysis (*F*_(3, 12)_ = 5.31, *p*=0.0146; n = 4). **(C)** Bars correspond to densitometric analysis of level of synaptophysin/α-tubulin. Data were presented as the mean ± S.E.M. Two-way ANOVA followed by Dunnett’s post hoc analysis (*F*_(3, 12)_ = 23.84, *p*<0.0001; n = 4). **(D)** Immunofluorescence images of MAP2, and synaptophysin. MAP2 was used as dendritic marker. Scale bars, 20 μm. **(E)** Quantification of intensity of synaptophysin. Data were presented as the mean ± S.E.M. Two-way ANOVA followed by Dunnett’s post hoc analysis (*F*_(3, 33)_ = 40.67, *p<*0.001; n = 12). **p* < 0.05, ***p* < 0.01; ****p* < 0.001.

**
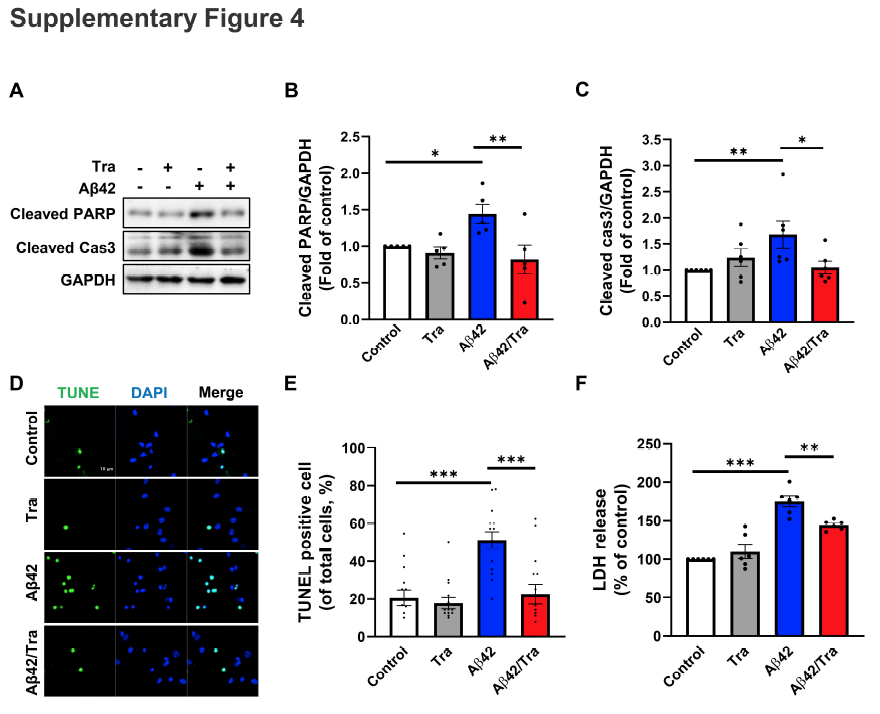
**

**Supplementary Figure 4. Trametinib protects the neuronal death.**

**(A)** Representative western blot analysis of cleaved PARP, and cleaved caspase 3 in SH-SY5Y cell. GAPDH was used as loading control. **(B)** Bars correspond to densitometric analysis of level of cleaved PARP/GAPDH. Data were presented as the mean ± S.E.M. Two-way ANOVA followed by Dunnett’s post hoc analysis (*F*_(3, 12)_ = 5.794, *p=*0.011; n = 5). **(C)** Bars correspond to densitometric analysis of level of cleaved caspase 3/GAPDH. Data were presented as the mean ± S.E.M. Two-way ANOVA followed by Dunnett’s post hoc analysis (*F*_(3, 15)_ = 5.026, *p=*0.0131; n = 6). **(D)** Immunofluorescence images of TUNEL staining of nuclei exhibiting DNA fragmentation in primary cortical neuron. **(E)** Bars correspond to quantification of TUNEL positive cells. Data were presented as the mean ± S.E.M. Two-way ANOVA followed by Dunnett’s post hoc analysis (*F*_(3, 42)_ = 11.46, *p<*0.0001; n = 15). **(F)** LDH release was measured from culture medium in primary cortical neuron. Data are shown as the percent of values in control value. Data were presented as the mean ± S.E.M. Two-way ANOVA followed by Dunnett’s post hoc analysis (*F*_(3, 15)_ = 46.1, *p<*0.0001; n = 6). **p* < 0.05, ***p* < 0.01; ****p* < 0.001.

**
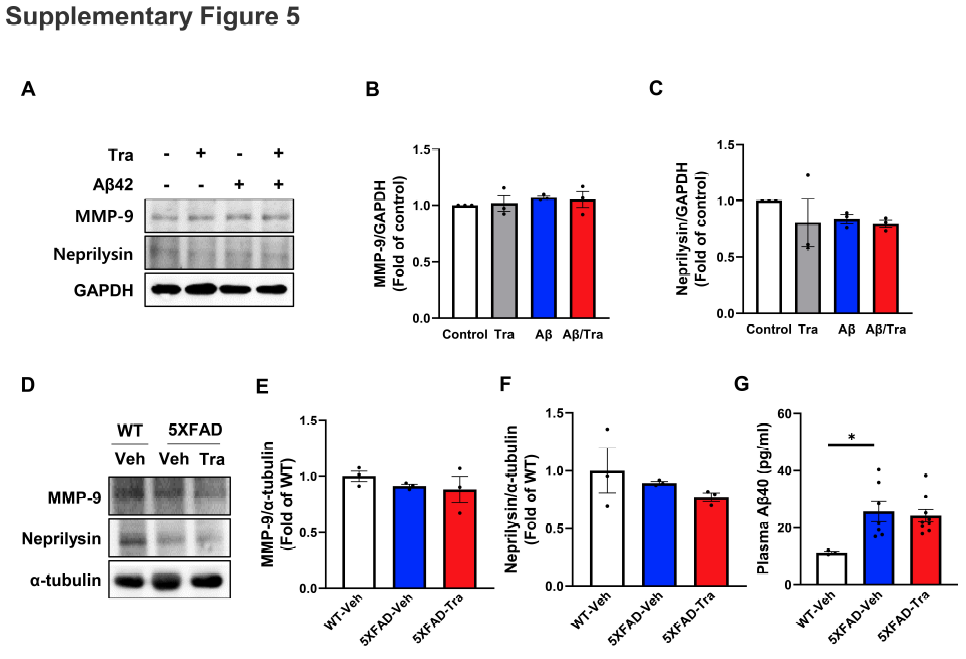
**

**Supplementary Figure 5. Trametinib do not alter the level of extracellular proteases and the plasma level of Aβ.**

**(A**) Representative western blot analysis of primary cortical neuron lysates for indicated proteins. GAPDH was used as loading control. **(B)** Bars correspond to densitometric analysis of level of MMP-9/GAPDH (*F*_(3,6)_ = 0.295, *p=*0.8281; n = 3). **(C)** Bars correspond to densitometric analysis of level of neprilysin/GAPDH (*F*_(3, 6)_ = 0.7272, *p=*0.572; n = 3). Statistical significance was determined using a two-way ANOVA followed by Dunnett’s post hoc analysis. **(D**) The 5-months 5XFAD mice were administered vehicle or 0.1 mg/kg of trametinib for 10 weeks by oral gavage once a day. Representative western blot analysis of 5XFAD cortex for indicated proteins. α-tubulin was used as loading control. **(E)** Bars correspond to densitometric analysis of level of MMP-9/α-tubulin (*F*_(2, 6)_ = 0.7089, *p=*0.5292; n = 3). **(F)** Bars correspond to densitometric analysis of level of neprilysin/α-tubulin (*F*_(2, 6)_ = 1.031, *p=*0.4123; n = 3). **(G)** The 9-month-old 5XFAD mice were administered either vehicle or 0.1 mg/kg of trametinib for 6 weeks by oral gavage once a day. The level of Aβ40 in plasma of 5XFAD mice was measured by ELISA (*F*_(2, 16)_ = 4.514, *p=*0.0279; WT- vehicle group; n = 3, 5XFAD-vehicle group; n = 7, 5XFAD-trametinib group; n = 9). Data were presented as the mean ± S.E.M. Statistical significance was determined using a one-way ANOVA followed by Dunnett’s post hoc analysis.

**
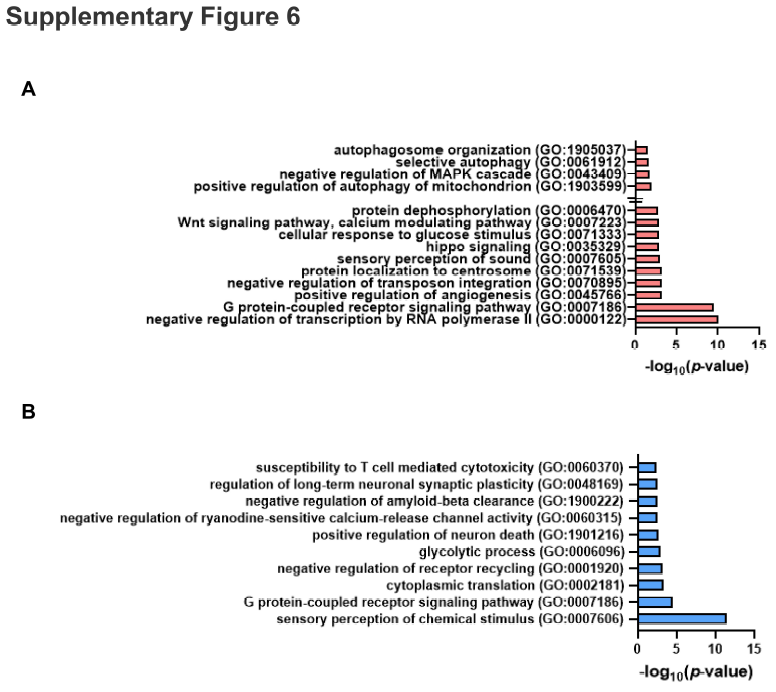
**

**Supplementary Figure 6. Gene ontology analysis from RNA-Seq of brains of the wild type C57BL/6 mice.**

**(A**, **B)** Up-regulated genes (**A**) and down-regulated genes (**B**) related with biological processes in bulk RNA-Seq from whole brains of the wild type C57BL/6 mice.


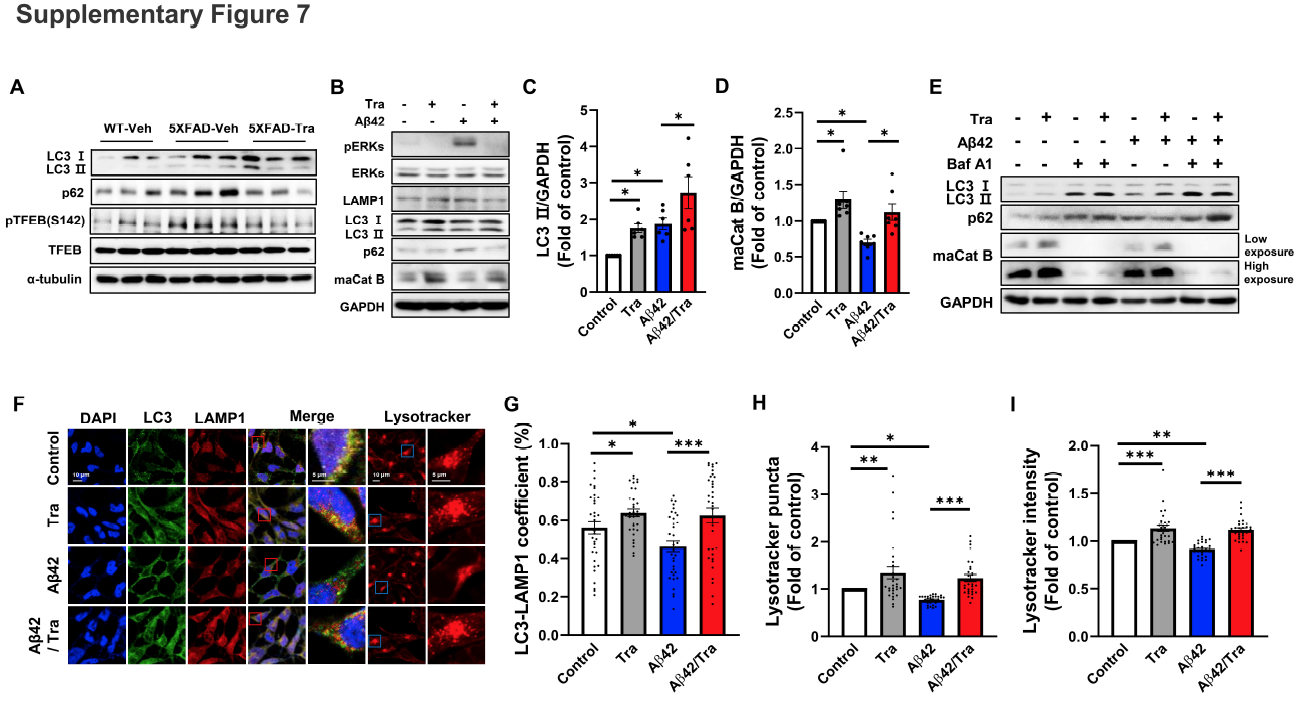


**Supplementary Figure 7. Trametinib increases autophagic flux in 5XFAD mice and SH-SY5Y cell.**

**(A)** Western blot analysis of 5XFAD mice brain cortex lysates for indicated proteins. **(B)** Representative western blot analysis of SH-SY5Y cell lysates for indicated proteins. GAPDH was used as loading control. **(C)** Bars correspond to densitometric analysis of level of LC3Ⅱ/GAPDH. Data were presented as the mean ± S.E.M. Two-way ANOVA followed by Dunnett’s post hoc analysis (*F*_(3, 15)_ = 13.02, *p=*0.0002; n = 6). **(D)** Bars correspond to densitometric analysis of level of cathepsin B/GAPDH. Data were presented as the mean ± S.E.M. Two-way ANOVA followed by Dunnett’s post hoc analysis (*F*_(3, 18)_ = 10.98, *p=*0.0003; n = 7). **(E)** Representative western blot analysis of LC3, p62, cathepsin B, and GAPDH. Cells were treated with trametinib with Aβ42 oligomer for 44 h, and then Baf A1 (100 nM) was added to the culture for 4 h. **(F)** Immunofluorescence images of LC3, and LAMP1. Images of lysotracker staining. Scale bars, 5 or 10 μm. **(G-I)** Quantification of the co-stained ratio with LC3 and LAMP1 **(G)** (*F*_(3, 99)_ = 9.875, *p<*0.0001; n = 34), number of lysotracker puncta **(H)** (*F*_(3, 76)_ = 11.82, *p<*0.0001; n = 37), and intensity of lysotracker **(I)** (*F*_(3, 84)_ = 27.27, *p<*0.0001; n = 29) in SH-SY5Y cells. Data were presented as the mean ± S.E.M. Two-way ANOVA followed by Dunnett’s post hoc analysis. **p* < 0.05, ***p* < 0.01; ****p* < 0.001.

**
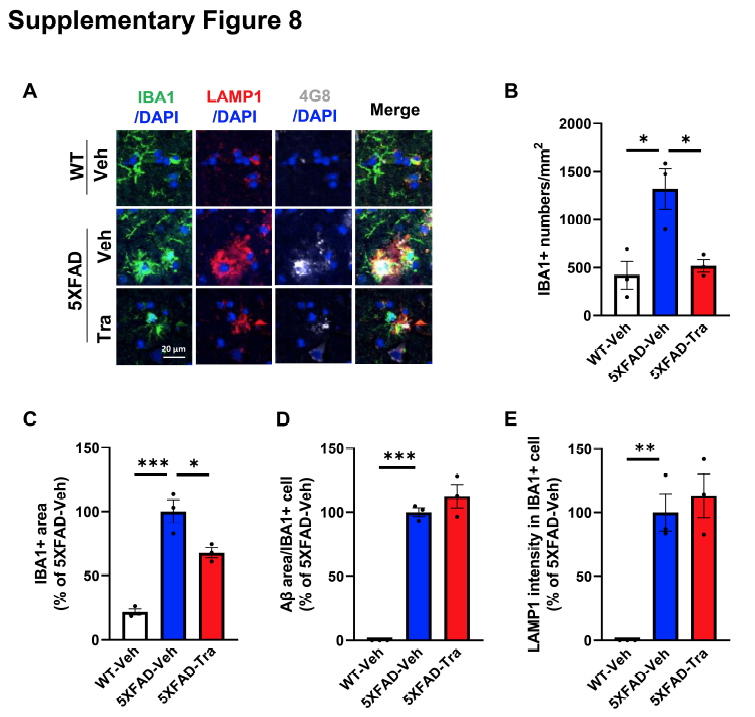
**

**Supplementary Figure 8. Changes in microglial activation and lysosomal activity by trametinib.**

The 9-month-old 5XFAD mice were administered either vehicle or 0.1 mg/kg of trametinib for 6 weeks by oral gavage once a day. **(A**) Immunofluorescence staining images of Aβ, LAMP1, and IBA1 in the cortex of 5XFAD mice. Scale bars, 20 μm. **(B)** Quantification of the number of IBA1^+^ microglia. Data were presented as the mean ± S.E.M. One-way ANOVA followed by Dunnett’s post hoc analysis (*F*_(2, 6)_ = 10.5, *p=*0.0114). **(C)** Quantification of area of Iba1^+^ microglia. Data were presented as the mean ± S.E.M. One-way ANOVA followed by Dunnett’s post hoc analysis (*F*_(2, 6)_ = 45.18, *p=*0.0002). **(D)** Quantification of the area of Aβ within IBA1^+^microglia area. Data were presented as the mean ± S.E.M. One-way ANOVA followed by Dunnett’s post hoc analysis (*F*_(2, 6)_ = 119.6, *p<*0.0001). **(E)** Quantification of LAMP1 intensity within IBA1^+^ microglia. Data were presented as the mean ± S.E.M. One-way ANOVA followed by Dunnett’s post hoc analysis (*F*_(2, 6)_ = 22.68, *p=*0.0016). **p* < 0.05; ***p* < 0.01; ****p* < 0.001.

**
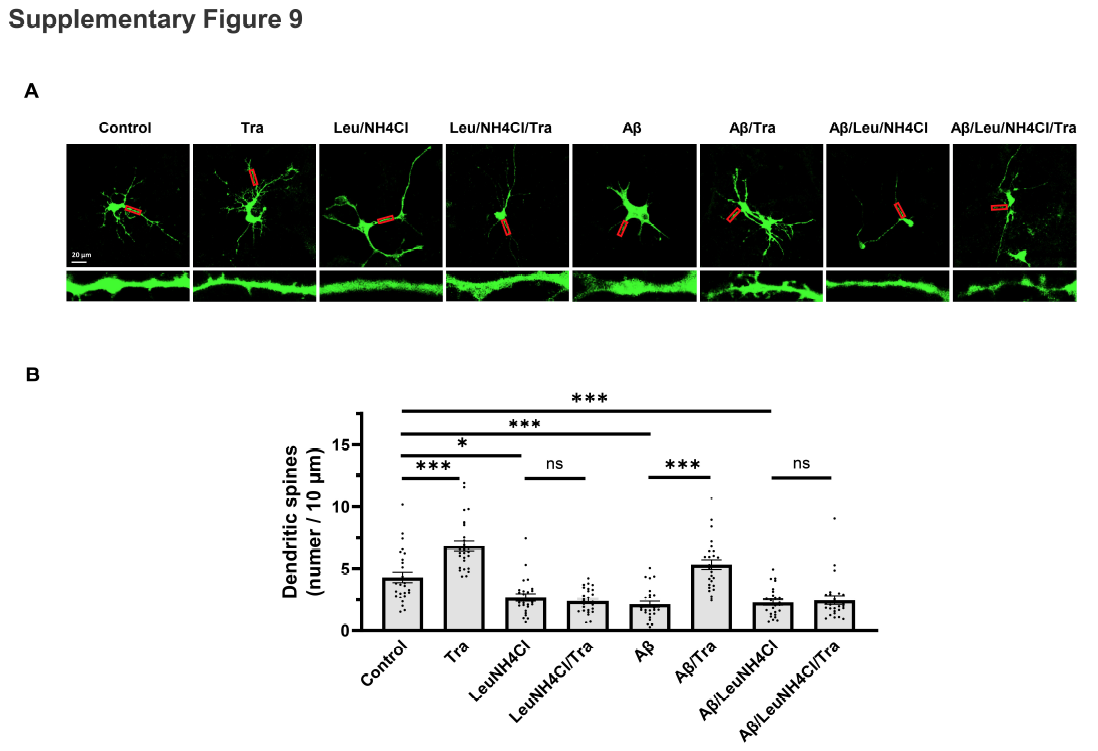
**

**Supplementary Figure 9. Lysosomal inhibitors block the protective effect of trametinib on Aβ42-induced-dendritic spine loss.**

Primary hippocampal neurons (DIV22) were transfected with GFP plasmid DNA, treated with 100 nM trametinib, 5 μM Aβ42 oligomer, and/or 20 μM leupeptin/ 10 mM NH_4_Cl for 48 hr, and dendritic spine density were measured. **(A**) Representative images of dendritic spine. Scale bars, 20 μm. **(B)** Quantification of data from **A.**  Data were presented as the mean ± S.E.M. Two-way ANOVA followed by Dunnett’s post hoc analysis. (*F*_(7, 175)_ = 30.07, *p<*0.0001; n = 26). **p* < 0.05; ****p* < 0.001.

**
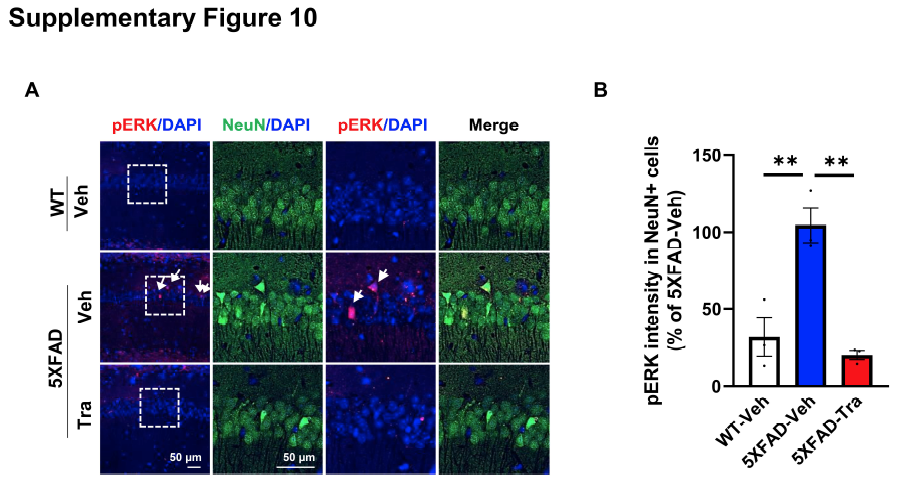
**

**Supplementary Figure 10. Trametinib decreases ERK activation in neuron of 5XFAD mice.**

The 5-month-old 5XFAD mice were administered either vehicle or 0.1 mg/kg of trametinib for 10 weeks by oral gavage once a day. **(A)** Immunofluorescence staining images of pERK and NeuN in the hippocampus CA1 of 5XFAD mice. Scale bars, 50 μm. **(B)** Quantification of pERK level within NeuN+ neurons. Data were presented as the mean ± S.E.M. One-way ANOVA followed by Dunnett’s post hoc analysis. (*F*_(2, 6)_ = 21.27, *p=*0.0019; n = 3). ***p* < 0.01.

**
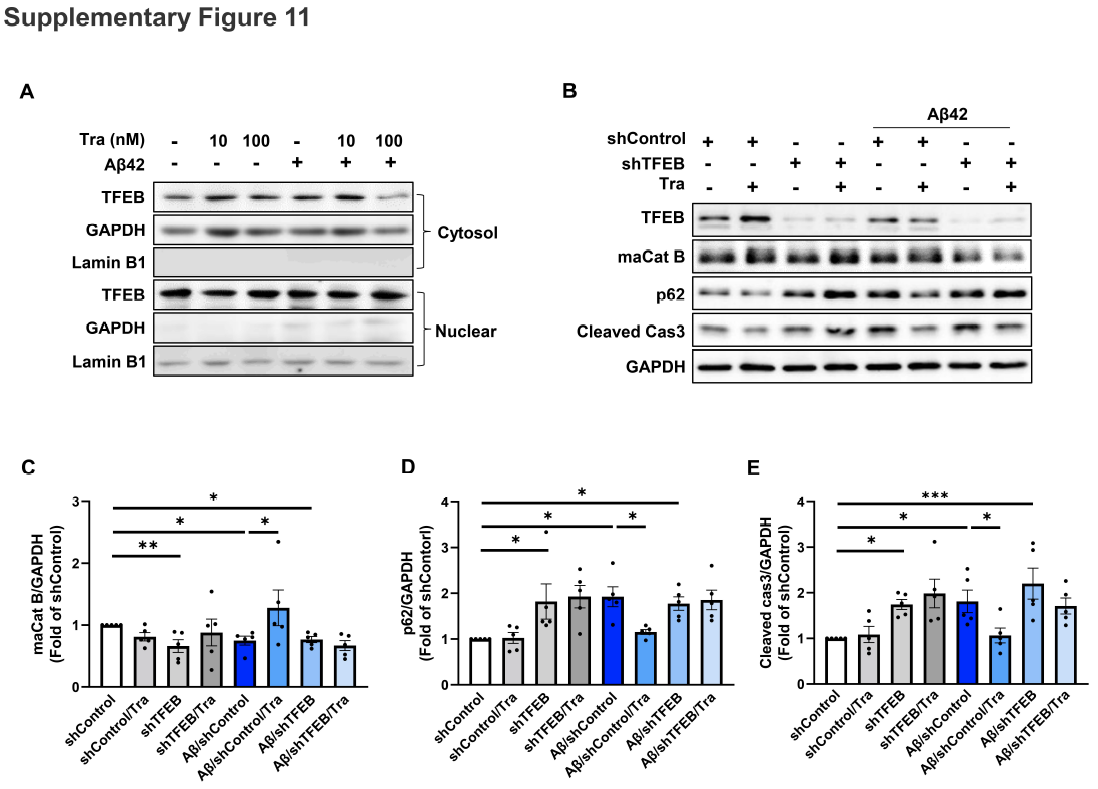
**

**Supplementary Figure 11. TFEB knockdown prevents the autophagic lysosomal activation and neuroprotective effect of trametinib.**

**(A)** Representative western blot analysis of TFEB in the cytosolic and nuclear fractions of SH-SY5Y cell. GAPDH and lamin B1 were used as cytosolic and nuclear fractions marker, respectively. Three independent experiments were performed. **(B)** Representative western blot analysis of primary cortical neuron lysates for indicated proteins. GAPDH was used as loading control. **(C)** Bars correspond to densitometric analysis of level of cathepsin B/GAPDH. Data were presented as the mean ± S.E.M. Two-way ANOVA followed by Dunnett’s post hoc analysis (*F*_(7, 28)_ = 3.083, *p=*0.0154; n = 5). **(D)** Bars correspond to densitometric analysis of level of p62/GAPDH. Data were presented as the mean ± S.E.M. Two-way ANOVA followed by Dunnett’s post hoc analysis (*F*_(7, 28)_ = 4.742, *p=*0.0013; n = 5). **(E)** Bars correspond to densitometric analysis of level of cleaved caspase 3/GAPDH. Data were presented as the mean ± S.E.M. Two-way ANOVA followed by Dunnett’s post hoc analysis (*F*_(7, 28)_ = 6.186, *p=*0.0002; n = 5). **p* < 0.05; ***p* < 0.01; ****p* < 0.001.

**
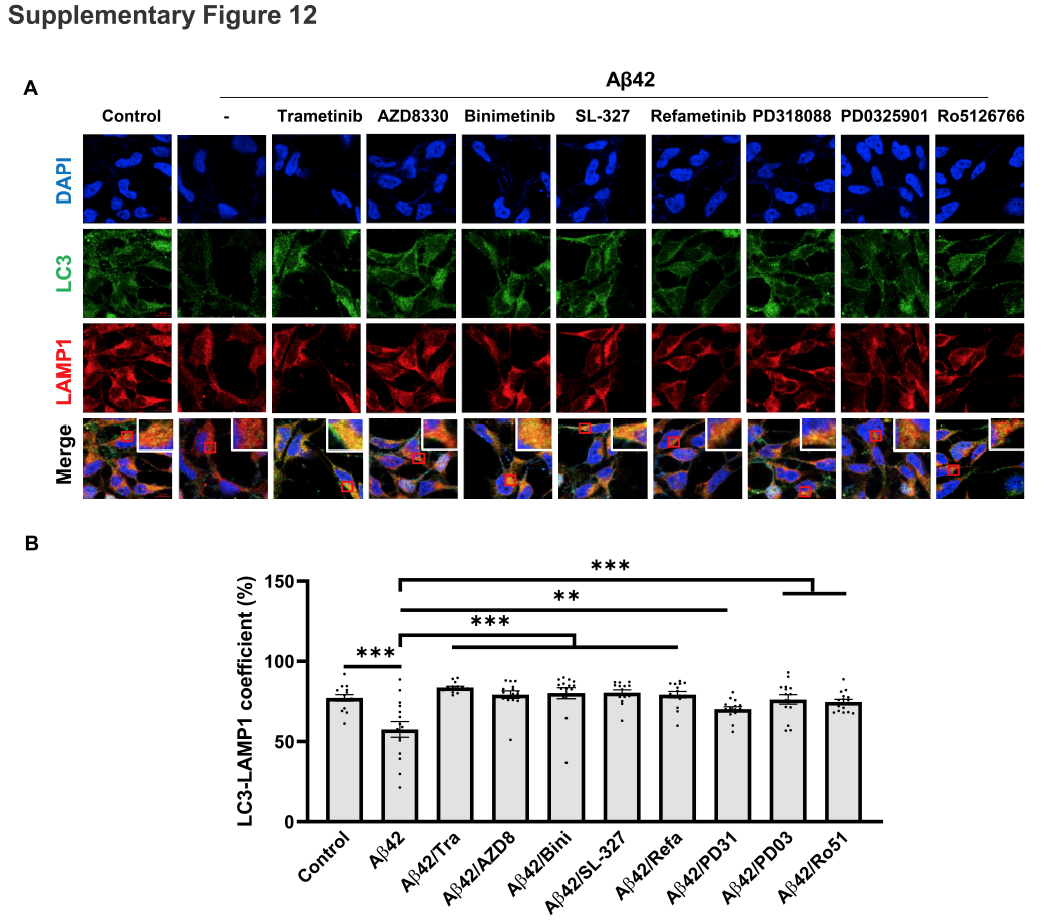
**

**Supplementary Figure 12. MEK inhibitors increase the autophagosome and lysosome fusion in SH-SY5Y cell.**

**(A)** Immunofluorescence images of LC3 and LAMP1. Cells were treated with MEK inhibitors (trametinib, AZD8330, binimetinib, SL-327, refametinib, PD318088, PD0325901, and Ro5126766) with Aβ42 oligomer for 48 h. **(B)** Quantification of the co-stained ratio with LC3 and LAMP1. Data were presented as the mean ± S.E.M. Two-way ANOVA followed by Dunnett’s post hoc analysis (*F*_(9, 126)_ = 8.217, *p<*0.0001; n = 15). ***p* < 0.01; ****p* < 0.001.


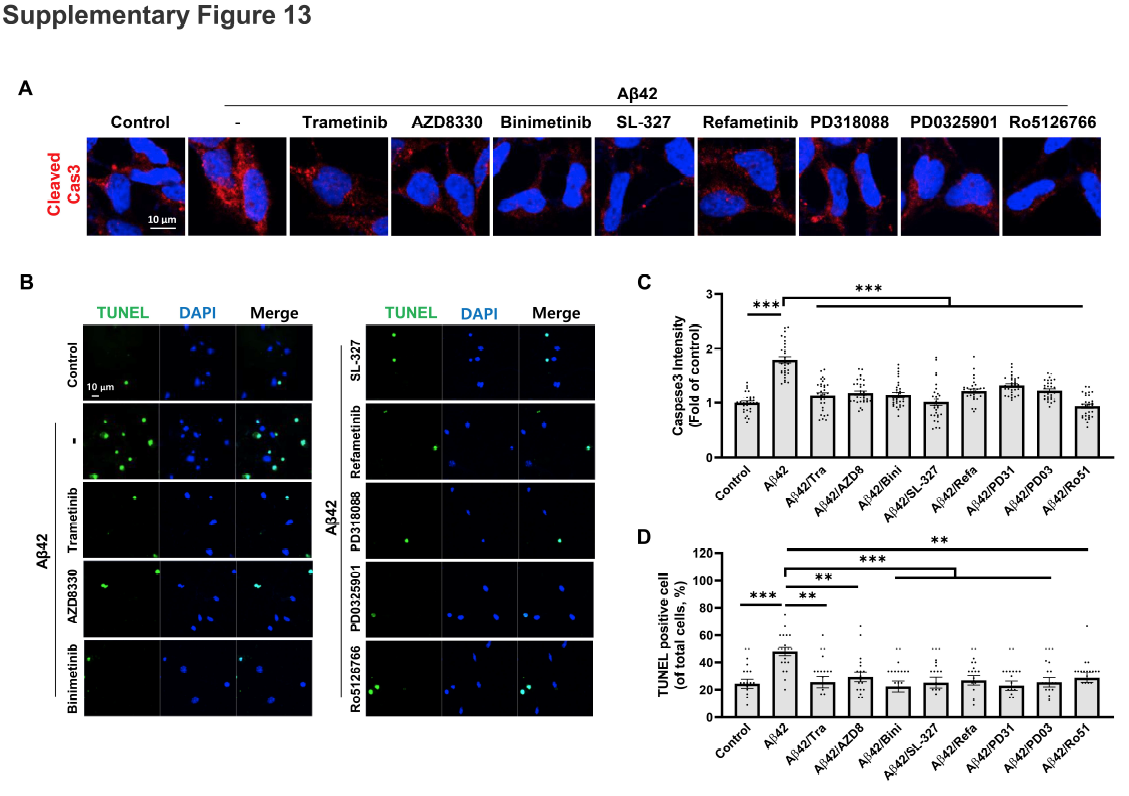


**Supplementary Figure 13. MEK inhibitors protects neuronal cells from Aβ42-induced death.**

**(A)** Immunofluorescence images of cleaved caspase 3 in SH-SY5Y cells. Scale bars, 10 μm. **(B)** Immunofluorescence images of TUNEL staining of nuclei exhibiting DNA fragmentation in primary cortical neuron. **(C)** Quantification of intensity of caspase-3. Data were presented as the mean ± S.E.M. Two-way ANOVA followed by Dunnett’s post hoc analysis (*F*_(9, 290)_ = 29.77, *p<*0.0001; n = 30). **(D)** Bars correspond to quantification of TUNEL positive cells. Data were presented as the mean ± S.E.M. Two-way ANOVA followed by Dunnett’s post hoc analysis (*F*_(9, 171)_ = 4.125, *p<*0.0001; n = 20). ***p* < 0.01; ****p* < 0.001.


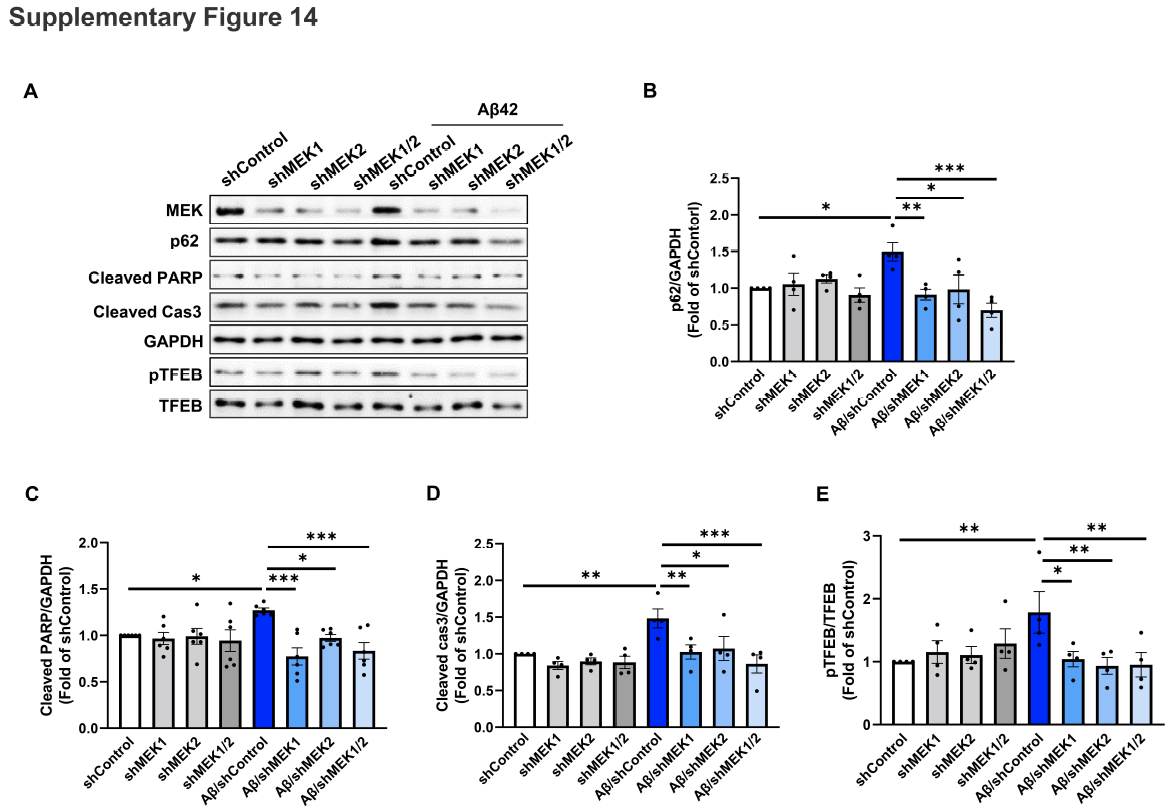


**Supplementary Figure 14. MEK knockdown increases autophagic lysosomal activity and protects primary neurons from Aβ-induced death.**

**(A)** Representative western blot analysis of primary cortical neuron lysates for indicated proteins. GAPDH was used as loading control. **(B)** Bars correspond to densitometric analysis of level of p62/GAPDH. Data were presented as the mean ± S.E.M. Two-way ANOVA followed by Dunnett’s post hoc analysis (*F*_(7, 21)_ = 4.997, *p=*0.0019; n = 4). **(C)** Bars correspond to densitometric analysis of level of cleaved PARP/GAPDH. Data were presented as the mean ± S.E.M. Two-way ANOVA followed by Dunnett’s post hoc analysis (*F*_(7, 35)_ = 4.799, *p=*0.0007; n = 6). **(D)** Bars correspond to densitometric analysis of level of cleaved caspase 3/GAPDH. Data were presented as the mean ± S.E.M. Two-way ANOVA followed by Dunnett’s post hoc analysis (*F*_(7, 21)_ = 5.903, *p=*0.0007; n = 4). **(E)** Bars correspond to densitometric analysis of level of pTFEB/TFEB. Data were presented as the mean ± S.E.M. Two-way ANOVA followed by Dunnett’s post hoc analysis (*F*_(7, 21)_ = 3.414, *p=*0.0135; n = 4). **p* < 0.05; ***p* < 0.01; ****p* < 0.001.

**Supplementary Tables**

**Table S1. Sources of human brain lysate**

| **Number** | **Diagnosis** | **Age (y)** | **Sex** | **Tissue** | **Manufacturer, Cat No.** |
| --- | --- | --- | --- | --- | --- |
| 1 | Normal control | 82 | Male | Brain | Genetex, GTX28771 |
| 2 | Normal control | 85 | Male | Brain | MYBioSource, MBS151732 |
| 3 | Normal control | 82 | Male | Brain | BioChain, P124035 |
| 4 | Normal control | 77 | Male | Brain | BioChain, P124035-DC |
| 5 | Alzheimer's Disease | 75 | Male | Brain | Genetex, GTX26622 |
| 6 | Alzheimer's Disease | 83 | Male | Brain | BioChain, P1236035Alz |
| 7 | Alzheimer's Disease | 87 | Male | Brain | MYBioSource, MBS657525 |
| 8 | Alzheimer's Disease | 93 | Female | Brain | BioChain, P1236035Alz-D02 |
| 9 | Alzheimer's Disease | 87 | Female | Brain | BioChain, P1236035Alz-D02 |

**Table S2. Antibodies commercial information**

| **Antibodies type** | **Designation** | **Manufacturer, Cat No.** |
| --- | --- | --- |
| Primary antibody/IHC, ICC | MAP2 | Millipore, MAB3418 |
| Primary antibody/IHC | phosphorylated neurofilament heavy subunit (pNFh) | Biolegend, 835503 |
| Primary antibody/IHC, ICC, WB | active caspase 3 | Cell signaling, 9664 |
| Primary antibody/IHC, ICC, WB | LAMP1 | Abcam, ab25245 |
| Primary antibody/IHC, ICC, WB | LC3 | Cell signaling, 3868 |
| Primary antibody/IHC | pERK | Abcam, ab214036 |
| Primary antibody/IHC | NeuN | Millipore, MAP377 |
| Primary antibody/IHC | SMI31 | Biolegend, 801601 |
| Primary antibody/IHC | IBa1 | Genetex, GTX100042 |
| Primary antibody/IHC | Aβ (clone 4G8) | Biolegend, 800704 |
| Primary antibody/ICC, WB | synaptophysin | Thermo, MA5-14532 |
| Primary antibody/WB | pERK | Cell signaling 4370 |
| Primary antibody/WB | ERK | Cell signaling, 4695 |
| Primary antibody/WB | PSD-95 | Thermo, MA1-046 |
| Primary antibody/WB | cathepsin B | Cell signaling, 31718  R&D system, MAB965 |
| Primary antibody/WB | p62 | Cell signaling, 5114  Abcam, ab56416 |
| Primary antibody/WB | TFEB | Biolegend, 852502 |
| Primary antibody/WB | pTFEB | Affinity, AF3845 |
| Primary antibody/WB | APP (C-terminal) | Sigma, A8717 |
| Primary antibody/WB | α-tubulin | Santa Cruz, sc-5286 |
| Primary antibody/WB | cleaved PARP | Cell signaling, 9548 |
| Primary antibody/WB | MEK | Cell signaling, 9122 |
| Primary antibody/WB | Lamin B1 | Cell signaling, 12586 |
| Primary antibody/WB | GAPDH | Cell signaling, 2118 |
| Secondary antibody/IHC/ICC | Alexa Fluor 488-conjugated anti-mouse IgG | Thermo, a21121 |
| Secondary antibody/IHC | Alexa Fluor 555-conjugated anti-mouse IgG | Thermo, a21422 |
| Secondary antibody/IHC | Alexa Fluor 488-conjugated anti-rat IgG | Thermo, a21208 |
| Secondary antibody/IHC/ICC | Alexa Fluor 555-conjugated anti-rabbit IgG | Thermo, a21428 |
| Secondary antibody/ICC | Alexa Fluor 555-conjugated anti-rat IgG | Thermo, a21434 |
| Secondary antibody/ICC | Alexa Fluor 488-conjugated anti-rabbit IgG | Thermo, a21206 |
| Secondary antibody/WB | HRP-conjugated goat anti-rabbit IgG | Thermo, 31460 |
| Secondary antibody/WB | HRP-conjugated goat anti-mouse IgG | Thermo, 31430 |
| Secondary antibody/WB | HRP-conjugated goat anti-rat IgG | Thermo, 31470 |
| Secondary antibody/WB | HRP-conjugated rabbit anti-goat IgG | Thermo, 31402 |

**Table S3. Primer list for qRT-PCR in mice**

| **Genes** | **Primer sequence (5’ → 3’)** |
| --- | --- |
| Ctsb | F: AGTCAACGTGGAGGTGTCTGCT |
|  | R: GTAGACTCCACCTGAAACCAGG |
| Ctsf | F: CACAGCTCAGTATGGGATCACC |
|  | R: TTGGCTGGACTCATCTTCCTGC |
| Atp6v1d | F: AGGAGCACAGACTGGTCGAAAC |
|  | R: CTCAGCCAATGAGAAGGCAGCT |
| Atp6v1h | F: GTTGCTGCTCACGATGTTGGAG |
|  | R: TGTAGCGAACCTGCTGGTCTTC |
| Map1lc3a | F: CTGCCTGTCCTGGATAAGACCA |
|  | R: CTGGTTGACCAGCAGGAAGAAG |
| Vps8 | F: GATGGACCATCTCCTGAAACAGG |
|  | R: AGCCTTCCTCTTGCTGACATCC |
| Sqstm1 | F: GCTCTTCGGAAGTCAGCAAACC |
|  | R: GCAGTTTCCCGACTCCATCTGT |
| Becn1 | F: CAGCCTCTGAAACTGGACACGA |
|  | R: CTCTCCTGAGTTAGCCTCTTCC |
| Uvrag | F: CAAGCTGACAGAAAAGGAGCGAG |
|  | R: GGAAGAGTTTGCCTCAAGTCTGG |
| Wipi1 | F: GAGTTCTGTGGAGCAGCTTGAC |
|  | R: GACGTTCATCTGCCGAGGTTTTG |
| Gapdh | F: CGTGCCGCCTGGAGAAACC |
|  | R: TGGAAGAGTGGGAGTTGCTGTTG |

**Table S4. Pharmacokinetic parameters of trametinib in the brain and plasma of ICR mice**

| **Dose**  **(mg/kg)** | **Brain** | | | **Plasma** | | | **Brain/**  **Plasma**  **ratio** |
| --- | --- | --- | --- | --- | --- | --- | --- |
|  | **AUC_t_**  **(ng·hr/g)** | **C_max_**  **(ng/g)** | **T_max_**  **(hr)** | **AUC_t_**  **(ng·hr/ml)** | **C_max_**  **(ng/ml)** | **T_max_**  **(hr)** |  |
| **0.05** | 60.95  ± 4.85 | 1.14  ± 0.06 | 33.60  ± 10.04 | 94.98  ± 13.56 | 3.14  ± 0.73 | 4.40  ± 3.29 | 0.64 |
| **0.2** | 291.89  ± 21.07 | 5.28  ± 0.55 | 36  ± 8.49 | 553.74  ± 68.64 | 49.79  ± 15.32 | 2.0  ± 0.0 | 0.53 |
| **0.8** | 1500.16  ± 186.47 | 33.99  ± 5.71 | 11.20  ± 7.16 | 3145.96  ± 735.39 | 367.79  ± 106.77 | 2.4  ± 0.89 | 0.48 |

**Table S5. Please see separated Excel file.**

**Table S6. Please see separated Excel file.**

**Table S7. 29 autophagic lysosomal genes upregulated by trametinib in RNA-Seq analysis**

| **Gene** | **Function** | **Location** | **Category** | **Reference** |
| --- | --- | --- | --- | --- |
| Clcn5 | Transporter | Cell membrane, endosome, membrane | Lysosomal membrane | [1] |
| Cubn | Receptor | Cell membrane, endosome, lysosome, membrane | Lysosome lumen | [2] |
| Atp6v0c | Transport | Membrane | Lysosomal acidification | [3] |
| Foxo3 | Transcription factors | Cytoplasm, membrane, nucleus | Autophagy regulation | [4] |
| Cd1d1 | Protein binding | Cell membrane, endosome, membrane | Lysosome | [5] |
| Chpt1 | Transferase | Cytoplasmic vesicle, membrane | Autophagosome formation | [6] |
| Abca9 | Transport | ER, membrane |  | [7] |
| Tlr7 | Receptor | ER, endosome, lysosome, membrane | Lysosome | [8] |
| Abca5 | Transport | Golgi, endosome, lysosome, membrane | Lysosome | [9] |
| Irgm2 | Hydrolase | Cell membrane, membrane | Autophagy assembly | [10] |
| Hyal1 | Glycosidase, Hydrolase | Lysosome | Lysosome | [11] |
| Abca1 | Transport | Cell membrane, membrane | Lysosome | [12] |
| Lrba | Autophagy | cell membrane, endosome, lysosome, membrane | Lysosome | [13] |
| Ramp2 | Receptor | cytoplasmic vesicle, membrane | Lysosome | [14, 15] |
| Manba | Glycosidase, Hydrolase | cytoplasmic vesicle, membrane | Lysosome | [16] |
| Arsg | Hydrolase | Lysosome | Lysosome | [17] |
| Atg2b | Autophagy | Membrane | Autophagy assembly | [18] |
| Tpcn2 | Channel, Ion transport | Lysosome, membrane | Autophagy | [19] |
| Mtm1 | Hydrolase,  Protein phosphatase | cell membrane, membrane, endosome | Autophagy assembly | [20] |
| Epg5 | Protein binding | Lysosome | Autophagosome maturation | [21] |
| Znrf2 | Transferase | cell membrane, endosome, lysosome, membrane | Lysosome | [22] |
| Ctsf | Lysosomal cysteine protease | cell membrane, cytoplasmic vesicle, lysososme | Lysosome | [23] |
| Ctbs | Glycosidase, Hydrolase | Lysosome | Lysosome | [24] |
| Tmem74 | Protein binding | Lysosome, membrane | Autophagy | [25] |
| Cln5 | Protein binding | Lysosome, membrane | Lysosome | [26] |
| Rb1cc1 | Autophagy | cytoplasm, lysosome | Autophagy assembly | [27] |
| Litaf | DNA-binding, Transcription regulation | cell membrane, endosome, lysosome, membrane, Golgi, Nucleus | Lysosomal membrane, Autophagy formation | [28] |
| Uvrag | Protein binding | lysosome, endosome, ER, cytoplasmic vesicle | Autophagy | [29] |
| Wipi1 | Protein binding | membrane, cytoplasmic vesicle, endosome, Golgi | Autophagy assembly | [30] |

**References**

1. Christensen EI, Devuyst O, Dom G, Nielsen R, Van der Smissen P, Verroust P, et al. Loss of chloride channel ClC-5 impairs endocytosis by defective trafficking of megalin and cubilin in kidney proximal tubules. Proc Natl Acad Sci U S A. 2003;100**:**8472-7.

2. Perea-Gomez A, Cases O, Lelievre V, Pulina MV, Collignon J, Hadjantonakis AK, et al. Loss of Cubilin, the intrinsic factor-vitamin B12 receptor, impairs visceral endoderm endocytosis and endodermal patterning in the mouse. Sci Rep. 2019;9**:**10168.

3. Hohn A, Sittig A, Jung T, Grimm S, Grune T. Lipofuscin is formed independently of macroautophagy and lysosomal activity in stress-induced prematurely senescent human fibroblasts. Free Radic Biol Med. 2012;53**:**1760-9.

4. Milan G, Romanello V, Pescatore F, Armani A, Paik JH, Frasson L, et al. Regulation of autophagy and the ubiquitin-proteasome system by the FoxO transcriptional network during muscle atrophy. Nat Commun. 2015;6**:**6670.

5. De Silva AD, Park JJ, Matsuki N, Stanic AK, Brutkiewicz RR, Medof ME, et al. Lipid protein interactions: the assembly of CD1d1 with cellular phospholipids occurs in the endoplasmic reticulum. J Immunol. 2002;168**:**723-33.

6. Dupont N, Chauhan S, Arko-Mensah J, Castillo EF, Masedunskas A, Weigert R, et al. Neutral lipid stores and lipase PNPLA5 contribute to autophagosome biogenesis. Curr Biol. 2014;24**:**609-20.

7. Albrecht C, Viturro E. The ABCA subfamily--gene and protein structures, functions and associated hereditary diseases. Pflugers Arch. 2007;453**:**581-9.

8. Delgado MA, Elmaoued RA, Davis AS, Kyei G, Deretic V. Toll-like receptors control autophagy. EMBO J. 2008;27**:**1110-21.

9. Kubo Y, Sekiya S, Ohigashi M, Takenaka C, Tamura K, Nada S, et al. ABCA5 resides in lysosomes, and ABCA5 knockout mice develop lysosomal disease-like symptoms. Mol Cell Biol. 2005;25**:**4138-49.

10. Singh SB, Davis AS, Taylor GA, Deretic V. Human IRGM induces autophagy to eliminate intracellular mycobacteria. Science. 2006;313**:**1438-41.

11. Puissant E, Gilis F, Dogne S, Flamion B, Jadot M, Boonen M. Subcellular trafficking and activity of Hyal-1 and its processed forms in murine macrophages. Traffic. 2014;15**:**500-15.

12. Xiong T, Xu G, Huang XL, Lu KQ, Xie WQ, Yin K, et al. ATP-binding cassette transporter A1: A promising therapy target for prostate cancer. Mol Clin Oncol. 2018;8**:**9-14.

13. Martinez Jaramillo C, Trujillo-Vargas CM. LRBA in the endomembrane system. Colomb Med (Cali). 2018;49**:**236-43.

14. Morfis M, Christopoulos A, Sexton PM. RAMPs: 5 years on, where to now? Trends Pharmacol Sci. 2003;24**:**596-601.

15. Wu X, Song Y, Liu W, Wang K, Gao Y, Li S, et al. IAPP modulates cellular autophagy, apoptosis, and extracellular matrix metabolism in human intervertebral disc cells. Cell Death Discov. 2017;3**:**16107.

16. Lovell KL, Zhu M, Drummond MC, Switzer RC, 3rd, Friderici KH. Distribution and Severity of Neuropathology in beta-Mannosidase-Deficient Mice is Strain Dependent. JIMD Rep. 2014;13**:**73-81.

17. Kowalewski B, Lubke T, Kollmann K, Braulke T, Reinheckel T, Dierks T, et al. Molecular characterization of arylsulfatase G: expression, processing, glycosylation, transport, and activity. J Biol Chem. 2014;289**:**27992-8005.

18. Velikkakath AK, Nishimura T, Oita E, Ishihara N, Mizushima N. Mammalian Atg2 proteins are essential for autophagosome formation and important for regulation of size and distribution of lipid droplets. Mol Biol Cell. 2012;23**:**896-909.

19. Gomez-Suaga P, Luzon-Toro B, Churamani D, Zhang L, Bloor-Young D, Patel S, et al. Leucine-rich repeat kinase 2 regulates autophagy through a calcium-dependent pathway involving NAADP. Hum Mol Genet. 2012;21**:**511-25.

20. Fetalvero KM, Yu Y, Goetschkes M, Liang G, Valdez RA, Gould T, et al. Defective autophagy and mTORC1 signaling in myotubularin null mice. Mol Cell Biol. 2013;33**:**98-110.

21. Wang Z, Miao G, Xue X, Guo X, Yuan C, Wang Z, et al. The Vici Syndrome Protein EPG5 Is a Rab7 Effector that Determines the Fusion Specificity of Autophagosomes with Late Endosomes/Lysosomes. Mol Cell. 2016;63**:**781-95.

22. Hoxhaj G, Caddye E, Najafov A, Houde VP, Johnson C, Dissanayake K, et al. The E3 ubiquitin ligase ZNRF2 is a substrate of mTORC1 and regulates its activation by amino acids. Elife. 2016;5.

23. Turk V, Stoka V, Vasiljeva O, Renko M, Sun T, Turk B, et al. Cysteine cathepsins: from structure, function and regulation to new frontiers. Biochim Biophys Acta. 2012;1824**:**68-88.

24. Persichetti E, Klein K, Paciotti S, Lecointe K, Balducci C, Franken S, et al. Lysosomal di-N-acetylchitobiase-deficient mouse tissues accumulate Man2GlcNAc2 and Man3GlcNAc2. Biochim Biophys Acta. 2012;1822**:**1137-46.

25. Yu C, Wang L, Lv B, Lu Y, Zeng L, Chen Y, et al. TMEM74, a lysosome and autophagosome protein, regulates autophagy. Biochem Biophys Res Commun. 2008;369**:**622-9.

26. Best HL, Neverman NJ, Wicky HE, Mitchell NL, Leitch B, Hughes SM. Characterisation of early changes in ovine CLN5 and CLN6 Batten disease neural cultures for the rapid screening of therapeutics. Neurobiol Dis. 2017;100**:**62-74.

27. Jung CH, Jun CB, Ro SH, Kim YM, Otto NM, Cao J, et al. ULK-Atg13-FIP200 complexes mediate mTOR signaling to the autophagy machinery. Mol Biol Cell. 2009;20**:**1992-2003.

28. Bertolo C, Roa S, Sagardoy A, Mena-Varas M, Robles EF, Martinez-Ferrandis JI, et al. LITAF, a BCL6 target gene, regulates autophagy in mature B-cell lymphomas. Br J Haematol. 2013;162**:**621-30.

29. Liang C, Feng P, Ku B, Dotan I, Canaani D, Oh BH, et al. Autophagic and tumour suppressor activity of a novel Beclin1-binding protein UVRAG. Nat Cell Biol. 2006;8**:**688-99.

30. Proikas-Cezanne T, Takacs Z, Donnes P, Kohlbacher O. WIPI proteins: essential PtdIns3P effectors at the nascent autophagosome. J Cell Sci. 2015;128**:**207-17.
